# Temperature‐ and Light‐Regulated Liquid Crystal Smart Window for Dynamic Control of Daylight and Solar Heat in All‐Weather Conditions

**DOI:** 10.1002/anie.7998291

**Published:** 2026-06-20

**Authors:** Yang Zhang, Ying Wu, Wenxuan Liu, Xin Wang, Dirk J. Broer, Ben L. Feringa, Jiawen Chen

**Affiliations:** ^1^ SCNU‐UG International Joint Laboratory of Molecular Science and Display South China Normal University Guangzhou China; ^2^ International Academy of Optoelectronics at Zhaoqing South China Normal University Zhaoqing China; ^3^ Stratingh Institute for Chemistry University of Groningen Groningen the Netherlands; ^4^ Institute of Carbon Neutrality Zhejiang Wanli University Ningbo China; ^5^ Department of Chemical Engineering and Chemistry Eindhoven University of Technology Eindhoven the Netherlands

**Keywords:** liquid crystal smart window, molecular motor, photo‐responsive liquid crystal materials

## Abstract

Smart windows that function in response to external conditions provide a promising approach to reduce heating, ventilation, and air conditioning energy consumption. However, it remains a major challenge to develop a smart window with environmental adaptability, multiple working states, and most importantly, the ability of dynamic management of solar light and heat with a simple and versatile molecular‐designed material. Here, we present a temperature‐ and light‐regulated smart window based on the interplay of light‐driven molecular motors and liquid crystal (LC) polymers. The window can dynamically switch among three distinct working states: transparent, reflective, and scattering, depending on ambient temperature and solar light intensity. The fast switching of working states enables excellent modulation of visible light transmittance (Δ*T*_lum_ = 75.2%) and near‐infrared light transmittance (Δ*T*_NIR_ = 49.3%), showing effective management of daylight and solar heat gain indoors. Simulation of energy regulation demonstrates that the smart window significantly reduces energy demand for indoor cooling and, therefore, is suitable for cities with different climate conditions. Our system provides an attractive approach toward more effective smart windows for sustainable and energy‐efficient green buildings.

## Graphical abstract

An environmentally adaptive smart window based on the interplay of light‐driven molecular motors and liquid crystal (LC) polymers is developed. The window can dynamically switch among three distinct working states: transparent, reflective, and scattering, depending on ambient temperature and solar light intensity, enabling excellent modulation of visible light transmittance (Δ*T*_lum_ = 75.2%) and near‐infrared light transmittance (Δ*T*_NIR_ = 49.3%), showing effective management of daylight and solar heat gain indoor.

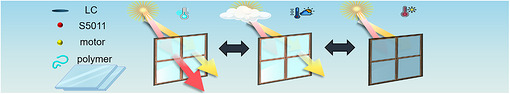

## 1 Introduction

Crucial steps against global climate change are major advances in energy efficiency and the reduction of carbon emissions [1, 2]. Buildings are responsible for 40% of global energy consumption and about 35% of greenhouse gas emissions, and therefore key targets for innovative materials to regulate energy use [3, 4]. Most of the consumed energies in buildings are related to indoor thermal management [5]. A conventional glass window provides good transmission of visible light but contributes little to reflection, or even blocking, of near‐infrared light, which is the main source of indoor heat gain, thereby increasing the demand for active cooling and enhancing energy consumption [6, 7].

To address the above inefficiency, smart windows have emerged with the requirement to dynamically modulate light and indoor heat in response to external conditions [8, 9, 10]. Considerable attention has been devoted to stimuli–responsive materials capable of autonomously switching between different working states. Electrical responsive systems have been developed, but they are under discussion as additional energy is required in order to maintain consistent voltages to keep the windows working [11]. Alternatively, temperature‐ and light‐triggered systems are promising due to their dynamic response to solar heat and ambient conditions [12, 13, 14]. Thermal‐ and photo‐responsive smart windows usually are based on materials such as liquid crystals (LCs) [15, 16], metal oxides [2, 17], ionic liquids [18, 19], hydrogels [20, 21], and perovskites [22, 23]. Among these, LC‐based smart windows have attracted significant attention. Because of their optical anisotropy and the ability to adjust internal molecular orientation, these materials provide versatile modulation of transparent, reflective, and scattering states without any, or only short‐pulsed, external energy input [24, 25]. For instance, azobenzene‐doped LC smart windows can automatically switch from a transparent state to a scattering state under UV irradiation, driven by the *trans*‐*cis* isomerization of azobenzene molecules, which disrupts molecular alignment of the LC [26, 27]. However, their performance is limited by the intrinsic *cis‐*to*‐trans* thermal relaxation of azobenzene molecules, which results in a gradual decrease in scattering over time [28, 29]. In addition, the need for irradiation with another wavelength of light for a fast reset of the original state of the system (visible light is usually required for *trans‐*to‐*cis* isomerization of azobenzene) further complicates the operation of the azobenzene‐based system [30]. Moreover, current photo‐responsive LC‐based smart windows typically switch between a high‐ and a low‐transmittance (opaque or reflective) state [31, 32, 33], which limits their capability toward more complicated weather conditions. For example, near‐infrared light is still required on a sunny day at temperatures below 20°C while most of the photo‐active smart windows are opaque under these conditions. Therefore, the development of an environment‐adaptative smart window with multiple working states would considerably contribute to a more economical future indoor climate management.

**FIGURE 1:**
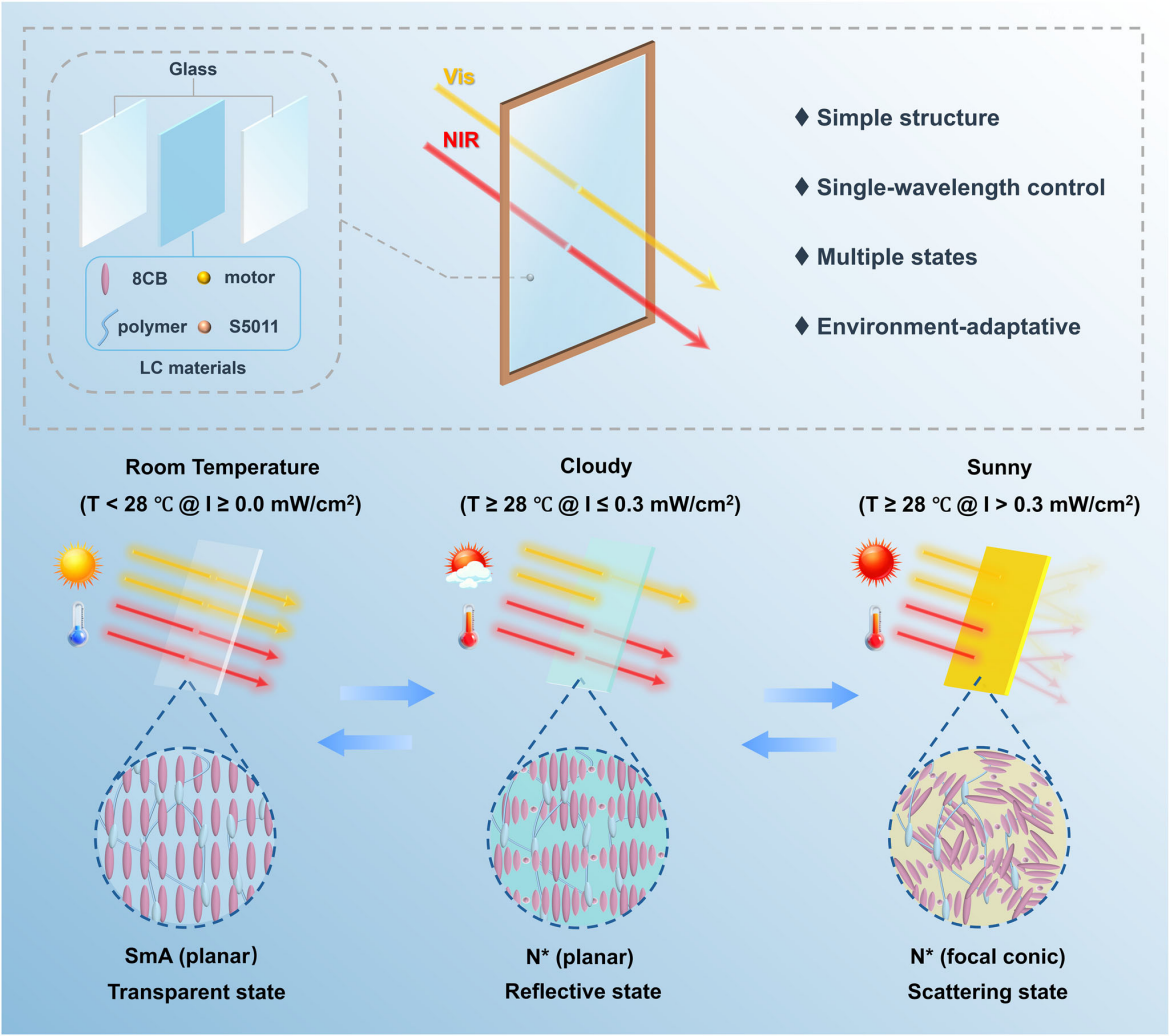
Representative scheme of a temperature‐ and light‐regulated liquid crystal smart window capable of reversibly switching among three working states in response to ambient temperature and sunlight intensity. The window is based on the interplay of light‐driven molecular motors and LC polymers. “T” represents temperature, and “I” represents UV light intensity.

Herein, we propose a temperature‐ and light‐regulated smart window based on light‐driven molecular motors and LC polymers. As shown in Figure 1, the window features a simple double‐glazing design, with an LC layer sandwiched between two glass substrates, making its preparation easier than that of a conventional multi‐layer smart window. The temperature‐ and light‐regulated liquid crystal (TLLC) smart window is able to reversibly switch among three distinct working states governed by ambient temperature and solar light intensity. At or below 28°C, the LC molecules adopt a smectic A (SmA) phase with planar alignment, allowing maximum transmission of visible and near‐infrared light. When the temperature rises above the SmA to chiral nematic (N*) transition temperature under low solar irradiation (≤ 0.3 mW/cm^2^), the light‐driven molecular motor and monomer S5011, acting as chiral dopants, induce the reorganization of the whole LC mixture into the N* phase, therefore reflecting part of the solar light. Under strong sunlight, the window becomes opaque and displays a light scattering state, thus providing maximum thermal shielding.

## 2 Results and Discussion

### 2.1 Photo‐Responsive Behavior of TLLC Smart Window

**FIGURE 2:**
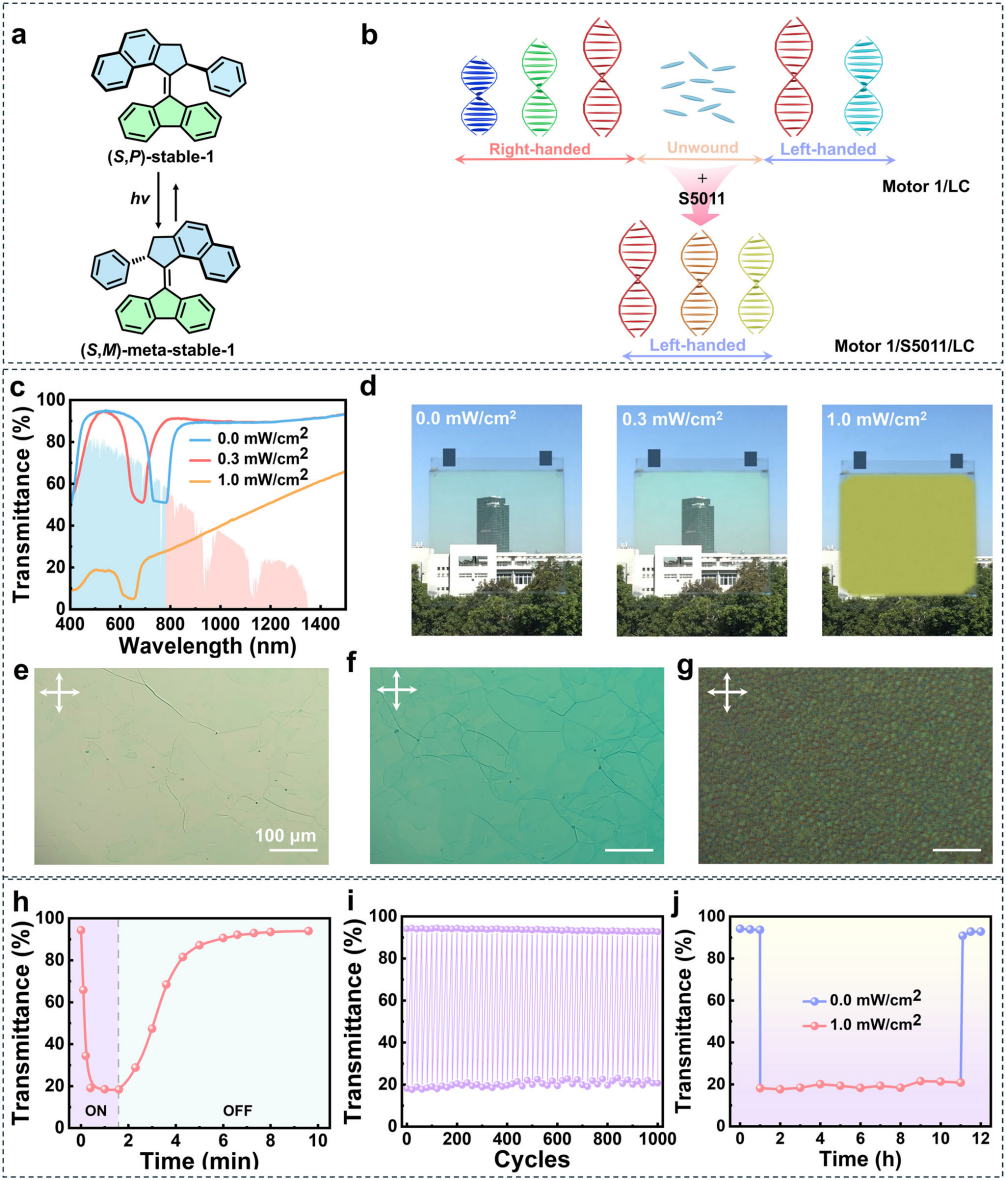
(a) Chemical structure and light‐driven rotary motion of chiral motor **1**. (b) UV‐induced pitch changes of CLCs doped with chiral motor **1** and its co‐doped system with S5011. (c)Transmittance spectrum and (d) photographs of TLLC smart window under irradiation with different UV intensities in the N* phase. (e–g) POM images of the TLLC smart window under UV irradiation at an intensity of (e) 0.0, (f) 0.3, and (g) 1.0 mW/cm^2^. (h) Response time, (i) cyclic, and (j) extensive UV irradiation test of a TLLC smart window, monitored by its transmittance at 550 nm.

To achieve an environment‐adaptative TLLC smart window with dynamically tunable transmittance, a photo‐responsive chiral dopant, that is, molecular motor **1**, was employed in the present study (Figure 2a). Motor **1** was prepared according to our previously reported procedures [34]. Both enantiomers of **1** were obtained by preparative chiral high‐performance liquid chromatography (Figure S1). In a 8CB LC host film, enantiomer (*S*,*P*)‐**1** exhibits right‐handed axial helicity with a helical twisting power (HTP) of +90 µm^−1^. Upon UV irradiation (*λ* = 365 nm), (*S*,*P*)‐**1** underwent a photo‐isomerization, resulting in the formation of (*S*,*M*)‐meta‐stable‐**1**, displaying a reversed axial chirality, that is, left‐handed helicity, and a negative HTP of ‐57 µm^−1^ was observed (Figures 2b and S2). UV–vis absorption spectra confirmed the photo‐isomerization, showing a shift of the maximum absorption from 390 to 415 nm due to the formation of *meta*‐stable isomer (Figure S3). The spectra can be fully regained when the sample is placed in the dark for 10 min, indicating the regeneration of (*S,P*)‐**1** by a thermal helix inversion step (Figure S3) [34].

After screening, a concentration of 0.3 wt% motor **1** was chosen as the optimal condition for preparation of the smart window, achieving a balance between photo‐responsive efficiency and relatively high transparency in the visible light region of the window (Figure S4). A 2.2 wt% of the nonresponsive chiral dopant S5011, which is able to induce left‐hand helicity of a cholesteric liquid crystal (CLC), was introduced to help tune the reflective band of the window to reach a wavelength range around 750–780 nm at a later stage. Finally, polymerizable monomers (1.0 wt%) were added to the LC matrix to enable fabrication of the TLLC smart window (For detailed procedures, see Supporting Information).

In order to systematically investigate the optical modulation performance of the prepared TLLC smart window under different UV light intensities, transmission spectra were measured. As both SmA and N phases can be formed in the 8CB LC host, measurements were conducted in each separated phase to assess the phase‐dependent optical response of the smart window. In the SmA phase, the window exhibited high optical transparency in the measured range (400–1500 nm), showing only slight changes under UV irradiation (Figure S5). Upon the transition from the Sm phase to the N* phase, the molecular order rearranges to form the helicoidal cholesteric texture (CLC phase) with the characteristic CLC reflection around 755 nm (Figure 2c, blue line). When exposed to UV irradiation with an intensity of 0.3 mW/cm^2^, the TLLC smart window gradually shows a homogenous, slightly pale‐blue color. A blueshift of the reflection band (Figure 2c, red line) from 755 to 674 nm was found while the remaining part of the visible spectrum kept its transmittance around 90.0%. Upon further increasing the UV intensity to 1.0 mW/cm^2^, the transmittances at all regions dropped dramatically, and the transmittance at 550 nm decreased from 94.2% to 18.5% specifically (Figure 2c, yellow line). In addition, enhanced light scattering was observed. Due to the strong scattering, the window displayed a yellowish and hazy appearance, which completely blocks objects behind the window (Figure 2d). Controlled experiments showed that windows containing only S5011 exhibited no optical modulation behavior under identical UV exposure conditions (Figure S6), which unambiguously indicates that light‐driven molecular motor **1** is key to the photo‐responsiveness of the prepared smart window.

To further understand how the chiral dopant motor **1** dynamically modulates the light transmittance of the TLLC window, polarized optical microscopy (POM) was employed. In the SmA phase, the highly symmetric LC structure minimized the impact of the rotary motion of motor **1** upon UV irradiation [35], resulting in negligible changes in optical transmittance under UV irradiation (Figure S5) and retaining the characteristic fan‐shaped texture under UV irradiation (Movie S1). When the temperature rises above 28°C, the LC mixture enters the N* phase and CLCs with a planar texture were formed in the presence of chiral dopant motor **1** and co‐dopant S5011 (Figure 2e). Upon UV irradiation with an intensity of 0.3 mW/cm^2^, photo‐triggered rotary motion of motor **1** took place, leading to the formation of *meta*‐stable‐**1** isomer with a reversed axial helicity (Figures 2a,b, S2, and S7). As the intensity of UV irradiation was moderate, only a portion of motor **1** was activated to rotate. As a result, the HTP due to the motor's induction decreased from +90 to +12 µm^−1^ (Figure S7), HTP of the whole LC moves toward a more left‐handed region, as co‐dopant S5011 possesses an intrinsic left‐handed axial chirality. The larger value of HTP shortens the helical pitch of the CLCs (Figure 2b) and therefore generates the corresponding blueshift in the reflective peak (Figure 2c). In addition, during the low‐intensity UV irradiation (0.3 mW/cm^2^), the texture of CLCs was found to remain in the planar state (Figure 2f and Movie S2). When the UV intensity was further increased to 1.0 mW/cm^2^, the fast rotary motion of all motor **1**, which leads to a completely reversed axial helicity of the CLCs, disrupts the stability of the planar texture, initiating a transition to the focal conic state (Figure 2c,g and Movie S3). Previous studies have shown that when motor **1** reaches its photo‐stationary state after photo‐isomerization, CLCs with *meta*‐stable‐**1** isomer can reassemble into a planar texture through molecular reorganization and form another CLC state [34, 36, 37]. However, in the present study, the introduction of a polymer network in TLLC smart window impeded the reassembly process by disrupting the ordered arrangement of the axis of CLCs, resulting in persistent light scattering and a pronounced reduction in transmittance of the system (Figures 2c,d and S8).

We next investigated the response time of the TLLC smart window under strong UV irradiation (1.0 mW/cm^2^). The transmittance at 550 nm (*T*_550nm_) was monitored, and it decreased rapidly from 94.2% to 18.5% within 18 s. After turning off the UV‐light, the transmittance was found to recover to 90.0% within 260 s and fully return to its initial state after 480 s (Figure 2h and Movie S3). Moreover, the transmittance at 550 nm showed no significant fatigue after 1000 cycles of UV irradiation (Figure 2i), indicating good stability of the smart window in correspondence with the high photochemical fatigue resistance of dopant **1** (Figure S16) [34, 37]. Furthermore, under extensive UV irradiation at an intensity of 1.0 mW/cm^2^ for 10 h, the TLLC window can maintain a low transmittance of around 20.0%. Upon ceasing the irradiation, the transparency of the window can recover to over 90.0% within 10 min (Figure 2j). An aging test was performed under conditions of 80°C, 80% relative humidity, and UV irradiation at an intensity of 5 mW/cm^2^ for 120 h. During the test, the optical performance of the window under different operating states remained largely stable (Figure S14). These results highlight the excellent performance of the TLLC smart window, which features the pronounced optical modulation (Δ*T*_550nm_ = 75.7%) ability and good stability, making it a promising candidate for future green building applications.

### 2.2 Thermal‐Responsive Behavior of TLLC Smart Window

**FIGURE 3:**
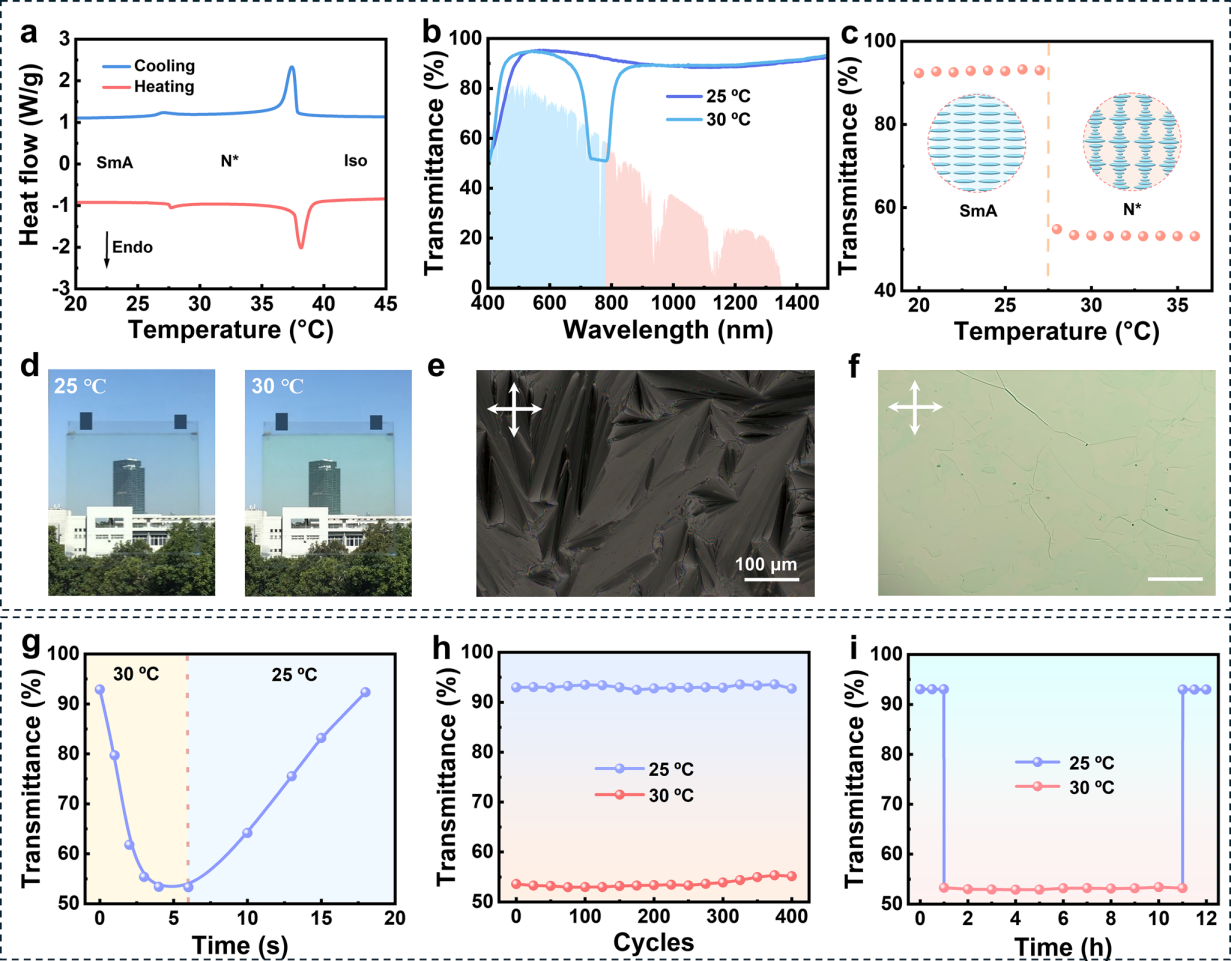
(a) DSC curves for the 8CB/**1**/S5011 mixtures. (b) Transmittance spectra of the TLLC smart window at 25°C (cyan) and 30°C (blue). (c) Variation of transmittance at 755 nm with different temperatures. (d) Photographs of TLLC smart window at 25°C and 30°C. (e,f) POM images of the TLLC smart window at (e) 25°C and (f) 30°C. (g) Response time, (h) cycling thermal stability, and (i) extensive heating tests of TLLC smart window under different thermal conditions, monitored by transmittance at 755 nm.

The above experimental data show that the TLLC smart window modulates its optical properties under different intensities of UV light irradiation. We then studied in detail the thermal‐responsive behavior of the TLLC smart window at different temperatures by differential scanning calorimetry (DSC). Pure LC monomer 8CB exhibits a SmA phase at 28.0°C, while the SmA‐N* and N*‐I transition temperatures are found at 33.8°C and 40.9°C, respectively (Figure S9). Noting that the clearing point of the LC mixture can be decreased by doping with a chiral dopant [38], therefore, in the present study, when motor **1** and S5011 were used, the 8CB/**1**/S5011 mixture showed SmA‐N* and N*‐I transition temperatures at 27.6°C and 38.2°C, respectively (Figure 3a).

Our TLLC smart window was found to adopt a SmA phase below 28°C, which exhibits high optical transparency in the range of 400–1500 nm (Figure 3b, cyan line). POM measurement showed a fan‐shaped texture of the LCs, confirming the planar alignment of the SmA phase (Figure 3e). Upon heating to the SmA‐N* transition temperature (28°C), chiral dopant motor **1** and S5011 triggered the formation of CLCs, as evidenced by the appearance of a reflective band centered at 755 nm (Figure 3b, blue line). This selective reflection is accompanied by the formation of a planar texture in the N* phase (Figure 3f). Due to the above phase transition and selective light reflection, the transmittance at the reflective wavelength (*T*_755nm_) dropped from 92.7% of the initial SmA phase to 53.2% of the N* phase at 28°C (Figure 3c), corresponding to the left‐handed reflection of circularly polarized light. Although CLC reflects one‐handedness of circularly polarized light, and it might not be able to maximally achieve effective solar heat modulation under unpolarized sunlight, a multilayer comprising two similar films with a half‐wave retardation film in between or scattering‐assisted configurations can improve the reflection of NIR. Overall, this results in a visible change of the TLLC window from a colorless transparent state to a slightly colored reflective state (Figure 3d). The decreased transmittance of 755 nm light effectively blocks a portion of the near‐infrared solar spectrum.

Next, we investigated responsive time of the TLLC smart window upon a change of temperature. Due to the fast phase transition of the LCs, the switch of window from transparent state to reflective state can be completed within a few seconds when the temperature rises above 28°C. Accordingly, decreasing the temperature below 28°C recovers the initial transparent state of the window within 12 s (Figure 3g). In addition, stability tests of the thermal modulation of the window were performed by alternating heating and cooling (Figure 3h). No significant fatigue was observed for the transmittance at 755 nm after 400 cycles (Figure 3h). Furthermore, after extensive heating above the SmA‐N* transition temperature for 10 h, the window maintained a stable transmittance of 53.0% at 755 nm, demonstrating excellent resistance to thermal degradation and good stability (Figure 3i).

### 2.3 Effect of Polymerizable Monomer Concentration on Responsive Performance of the TLLC Smart Window

**FIGURE 4:**
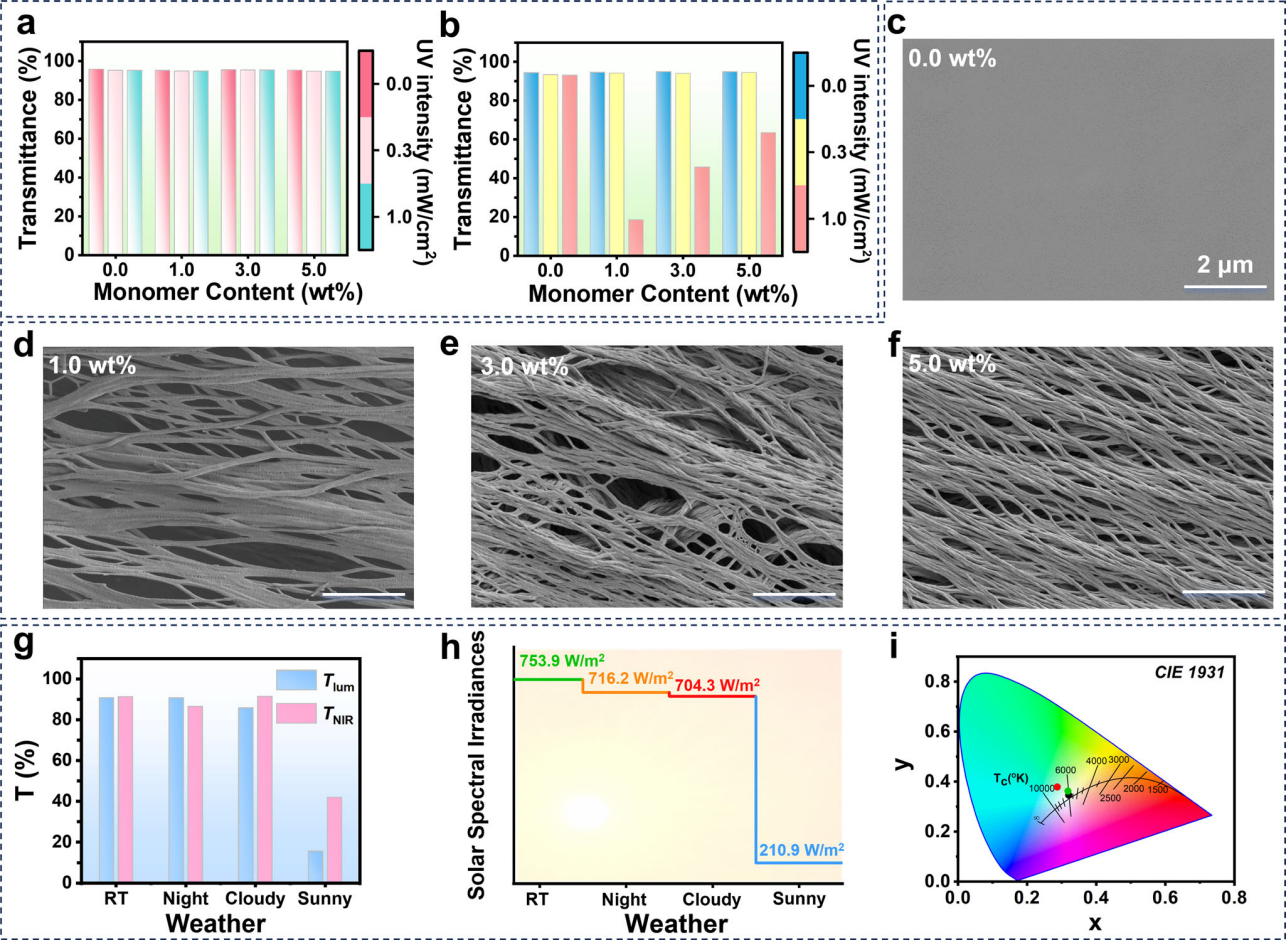
(a,b) Transmittance at 550 nm of TLLC smart windows prepared with varying monomer concentrations under UV irradiation at different intensities in (a) SmA phase and (b) N* phase. (c–f) SEM images of polymer network morphology in TLLC smart windows prepared with different monomer concentrations. (g) Visible light and near‐infrared light transmission in various weather conditions. (h) Solar irradiance entering the room and (i) CIE chromaticity coordinates of the TLLC smart window under different weather conditions.

The polymer network is known to play a crucial role in constructing LC‐based smart windows [39], we investigated the effect of polymerizable monomer concentration in the present study. At 25°C, when the LCs are in the SmA phase, TLLC smart windows prepared with different monomer concentrations, after polymerization, all exhibited a *T*_550nm_ of 94.4%, with little variation under irradiation at different UV light intensities (Figures 4a and S10). In this state, the smart window allows light to pass through with negligible disruption, keeping the natural daylight going indoors. When the LC mixture was brought to the N* phase (above 28°C), the pure CLC system (0.0 wt% monomer concentration) showed no significant change of the transmittance at 550 nm (*T*_550nm_ = 94.4%) (Figure 4b). In contrast, when 1.0, 3.0, and 5.0wt% monomers were employed, a significant decrease of *T*_550nm_ was observed under strong UV irradiation (1.0 mW/cm^2^) after polymerization, while moderate UV irradiation (0.3 mW/cm^2^) kept *T*_550nm_ intact (Figures 4b and S11). It is worthwhile to note that the addition of 1.0 wt% polymerizable monomers was found to provide the optimal condition, decreasing *T*_550nm_ from 94.4% to 18.5% upon strong UV irradiation (1.0 mW/cm^2^) (Figure 4b). The above decrease of transmittance under UV irradiation is attributed to phase separation induced by photo‐polymerization, which results in LC microregions separated by the polymer network. These networks disrupt the ordered arrangement of the helical axis after helical inversion of CLCs caused by the rotary motion of dopant **1**. No new stable CLC state could be formed, which usually happens for the system without polymer [34]. The texture of CLCs changed from planar to focal‐conic texture, therefore enhancing the light scattering. Scanning electron microscope (SEM) images of the TLLC smart window confirmed the phase separation induced by polymerization (Figure 4c–f). Furthermore, as the monomer concentration increases, the light‐modulation capability of the TLLC smart window gradually decreases. When the concentration of monomer was increased from 1.0 to 5.0 wt%, the pore area fraction dropped from 22.9% to 17.6%, whereas the pore number density rose from 3.4 to 15.2 pores/µm^2^, which demonstrates a gradual transition from a relatively sparse polymer network with large pores to a highly dense network structure. Although the dense polymer network formed at higher monomer concentrations exhibits an increased number of pores, the overall pore area fraction is reduced. A decrease of Δ*T*_550nm_ is found, from 75.7% to 31.5% under 1.0 mW/cm^2^ UV exposure (Figure 4b). This decrease is due to the fact that, when a denser polymer network is formed (5.0 wt%), it would restrict the rotary motion of motor **1**, which is key to the disorder of the LC system, thereby weakening the scattering modulation capability. Moreover, SEM reveals that the polymer network exhibits submicron‐scale pore sizes, and such structural inhomogeneities usually introduce spatial refractive index fluctuations in the system, thereby enhancing light scattering. For scattering centers with dimensions much smaller than the incident light wavelength, shorter wavelengths are scattered more strongly. Overall, the TLLC smart window fabricated with 1.0 wt% monomer demonstrated the most effective responsive performance and was therefore used for further studies.

### 2.4 Smart Window for Energy‐Efficient Building

The crucial property of a smart window for green building is the effective management of visible light and heat indoors under different conditions. The key to this management is the dynamic modulation of transmission of visible light (*T*_lum_) and near‐infrared (*T*_NIR_) light. The TLLC smart window, prepared with optimal performance, was investigated outdoors under different weather conditions and monitored by in situ optical measurement (Figures S13 and S15). The transmittance (*T*_lum_) in the visible range (380–780 nm), near‐infrared range (780–2500 nm) (*T*_NIR_), and solar range (300–2500 nm) (*T*_sol_) were then calculated using equation.

$$\;T_{\mathrm{lum}/\mathrm{NIR}/\mathrm{sol}}=\frac{\int \varphi_{\mathrm{lum}/\mathrm{NIR}/\mathrm{sol}}\left(\lambda \right)T\left(\lambda \right)d\lambda }{\int \varphi_{\mathrm{lum}/\mathrm{NIR}/\mathrm{sol}}\left(\lambda \right)d\lambda }$$

where *T*(λ) is the transmittance at wavelength 𝜆, φ_lum_(λ) is the standard luminous efficiency function of photosensitive vision, φ_NIR_(λ) is the AM1.5 solar irradiance spectrum in the near‐infrared region, and φ_sol_(λ) is the AM1.5 solar irradiance spectrum over the entire solar spectral range. Solar heat gain coefficient (SHGC) is calculated as well and summarized in Table S1. At or below 28°C, the window adopts a transparent state with *T*_lum_ = 90.8% and *T*_NIR_ = 91.3%, respectively. When the temperature is above 28°C at night (0.0 mW/cm^2^), *T*_lum_ is found to maintain at 90.8%, while *T*_NIR_ decreases to 86.5%, indicating that the TLLC window maintains high penetration of visible light while partially reducing near‐infrared light transmission. When it is on a cloudy day time above 28°C (UV intensity is lower than 0.3 mW/cm^2^), *T*_lum_ is further decreased to 86.0%. In addition, when it is a sunny day above 28°C (UV intensity is above 1.0 mW/cm^2^), the TLLC window presents a scattering state, where *T*_lum_ and *T*_NIR_ are further decreased to 15.6% and 42.0%, respectively (Figure 4g). Large contrast ratios (Δ*T*_lum_ = 75.2% and Δ*T*_NIR_ = 49.3%), were found (Figure 4g) when compared to the original transparent state. In addition, the indoor thermal modulation of the TLLC smart window was evaluated by calculating the total indoor irradiance under different conditions as well. As shown in Figure 4h, with the fast switching of working states of TLLC smart window, the total solar irradiance entering the room decreases significantly from 753.9 W/m^2^ below 28°C to 210.9 W/m^2^ on warm (> 28°C) sunny days. A comprehensive comparison between our work and representative smart window systems was summarized in Table S2, including various types of LC systems as well as typical smart window materials such as VO_2_, hydrogels, perovskites, and ionic liquids. The table clearly highlights the advantages of our system, including the broad solar spectral modulation capability, excellent long‐term durability, and most importantly, the absence of a need for continuous electrical driving. Besides thermal management, visual indication is another key factor for smart window applications [40, 41]. Based on the transmission spectrum of our TLLC smart window, chromaticity coordinates were calculated using the CIE 1931 diagram (Figure 4i). When the TLLC window is in the N* phase, its coordinates lie in the neutral gray region (0.32028 and 0.34607), indicating a colorless transparent appearance. As the UV intensity increases to 1.0 mW/cm^2^, the coordinates shift toward a higher color region (0.28726 and 0.37853), which shows a clear visible color. The obvious color change of the window can therefore serve as a good indicator for people indoors to be aware of the change of temperature and solar irradiation outdoors.

**FIGURE 5:**
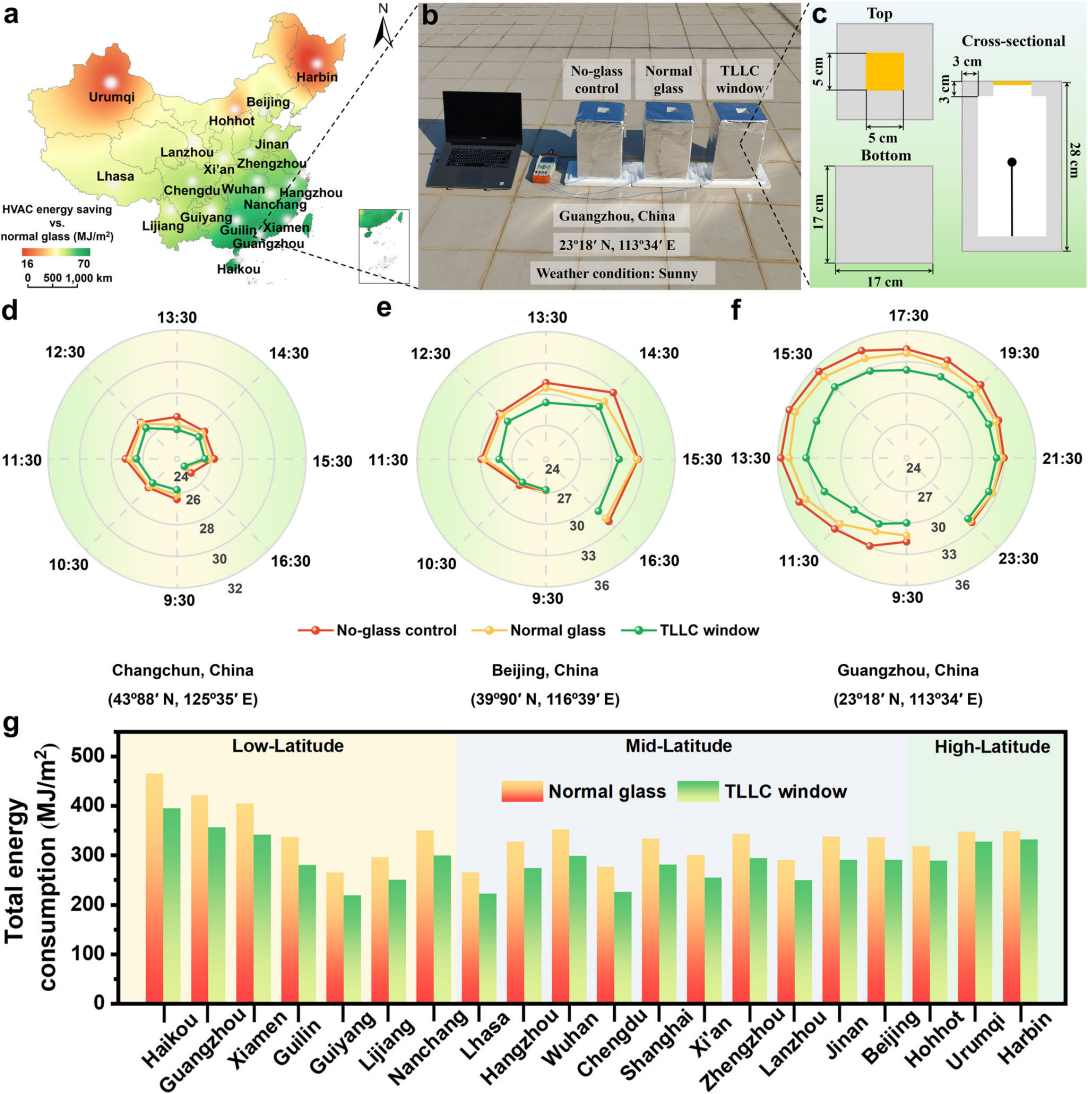
(a) Annual HVAC energy saving value for the TLLC smart window compared to that of a normal glass as the baseline in 20 different cities in China. (b) Photographs of the prepared testing equipment. (c) Dimensions and structures of the heat‐insulating box for the outdoor experiments. (d‐f) Internal temperature of the chamber for the energy‐saving test in (d) Changchun, (e) Beijing, and (f) Guangzhou. (g) Annual HAVC energy saving performance for normal glass and TLLC smart windows in each city.

With the above data in hand, three heat‐insulating boxes (external dimensions: 17 × 17 × 28 cm^3^; internal dimensions: 11 × 11 × 22 cm^3^; wall thickness: 3 cm) were used as test chambers under actual conditions (Figure 5a–c). The smart window sample was installed on the top of the box. The boxes were subsequently equipped with styrofoam panels and completely covered by aluminum foil. Styrofoam provided thermal insulation from the ambient environment, while the aluminum foils acted as reflective films to minimize external heating. The temperature inside the box was recorded via thermocouples. The experiments were conducted outdoors, including boxes with no glass (as a control experiment), normal glass, and TLLC window (sample size 5 × 5 cm^2^), respectively (Figure 5b). Outdoor experiments were conducted in three cities: Changchun (high‐latitude, 43°88′N), Beijing (mid‐latitude, 39°90′N), and Guangzhou (low‐latitude, 23°18′N). In Changchun (in May), the ambient temperature was below 26°C (Figure 5d). Under these conditions, the TLLC smart window remained in the SmA phase, maintaining full transparency and allowing most of the solar radiation into the chamber. Consequently, no significant difference in the internal temperature (Figure 5d) was observed for three setups (no window, normal window, and TLLC window). In contrast, the test conducted in Beijing exhibited the advantage of applying TLLC smart windows (Figure 5e). Before 10:30, the ambient temperature was around 27°C, and the internal temperatures of all three chambers were nearly identical. As the ambient temperature increased later in the day, the difference in internal temperature was observed. From 12:00 to 16:00, the internal temperature of the box equipped with TLLC smart window was on average 1.8°C lower than that with a normal window. Furthermore, the outdoor test conducted in Guangzhou, which is a city in a low‐latitude region, revealed a more pronounced thermal modulation effect. The ambient temperature of this city was above 30 °C throughout the day (in May). During the daytime (9:30–18:30), the chamber equipped with the TLLC smart window was found to have a temperature 2.3 °C lower than that with a normal window glass. A maximum 2.8°C difference in the internal temperature of three chambers was observed at 14:30 (Figure 5f). These results confirm that under solar irradiation and increasing ambient temperatures, the TLLC window can effectively scatter near‐infrared radiation, thereby reducing heat gain and contributing to passive cooling.

To further evaluate the potential of TLLC smart windows in buildings, an EnergyPlus‐based simulation model [42, 43] was developed for a prototype of a 10th‐floor building (40 × 40 × 40 m). Annual heating, ventilation, and air conditioning (HVAC) energy savings values were calculated using normal glass as the baseline [43]. The evaluations were run in 20 representative Chinese cities with different latitudes and climate zones. As shown in Figure 5g, the results demonstrate that the energy‐saving performance of the TLLC smart window is superior to that of normal glass. Most of the cities showed more than 50 MJ/m^2^ of the HVAC energy‐saving values, such as Haikou (70.2 MJ/m^2^), Guangzhou (64.4 MJ/m^2^), Guilin (57.1 MJ/m^2^), Nanchang (51.2 MJ/m^2^), Wuhan (53.1 MJ/m^2^), and Shanghai (52.1 MJ/m^2^). Interestingly, the average value of annual HVAC energy‐saving is 56.8 MJ/m^2^ in low‐latitude regions, which is 2.6 times higher than that (22.2 MJ/m^2^) in high‐latitude regions. It indicates that for cities in low‐latitude areas that have higher annual average ambient temperature, the TLLC smart window is more effective in managing the heat gain indoors. In addition, the energy‐saving effect is more pronounced from June to August, particularly in mid‐ and high‐latitude regions, where longer daytime and sunshine are common during summer (Figure S12). For example, the HVAC energy‐saving value in Beijing is around 12.0 MJ/m^2^ in spring (March–May), 13.1 MJ/m^2^ in autumn (September–November), and only 2.3 MJ/m^2^ in winter (December–February), whereas 18.2 MJ/m^2^ was found in summer. The above results highlight the excellent light and heat modulation performance of TLLC smart window across different seasons, confirming its good potential for application in real buildings.

## 3 Conclusion

In summary, we present here a TLLC smart window based on light‐driven molecular motor **1** and LC polymers that is able to autonomously modulate the transmission of visible and near‐infrared light. This responsive modulation is operated by fast switching of transparent, reflective, and scattering states of the LC mixture in the smart window, depending on the external condition. At RT (≤ 28°C), the window maintains high transparency (*T*_550nm_ = 94.2% and *T*_NIR_ = 89.7%). When the temperature exceeds 28°C under low UV intensity (≤ 0.3 mW/cm^2^), it converts to the N* phase and selectively reflects part of the solar light. Under strong sunshine (UV intensity > 0.3 mW/cm^2^) and higher temperature (> 28°C), the window adopts a scattering state and becomes hazy, providing a pronounced solar blocking effect with maximum Δ*T*_lum_ = 75.2% and Δ*T*_NIR_ = 49.3%, respectively. Field tests reveal that TLLC smart windows can reduce indoor temperature by up to 2.8°C in some cities, and annual HVAC energy simulations for 20 Chinese cities show a substantial energy‐saving effect, reaching a maximum 56.8 MJ/m^2^ in the low‐latitude region. For further scale‐up preparation, as our system still relies on the common layer‐by‐layer assembly strategy, we can take advantage of current manufacturing approaches such as large‐area coating of alignment layers, roll‐to‐roll coating, and lamination encapsulation. Our system represents a simple and versatile approach for the preparation of environmentally adaptive smart windows and demonstrates good performance toward the management of light and heat, offering a promising solution for energy‐efficient green buildings.

## Author Contributions

**Yang Zhang**: investigation. **Ying Wu**: investigation. **Wenxuan Liu**: investigation. **Xin Wang**: funding acquisition. **Dirk J. Broer**: writing – original draft. **Ben L. Feringa**: supervision, funding acquisition, writing – review and editing. **Jiawen Chen**: funding acquisition, conceptualization, supervision, writing – review and editing.

## Conflicts of Interest

The authors declare no conflict of interest.

## Supporting information




**Supporting File 1**: anie73186‐sup‐0001‐SuppMat.pdf.

**Supporting File 2**: anie73186‐sup‐0002‐MovieS.zip.

## Data Availability

The data that supports the findings of this study are available from the corresponding author upon reasonable request.
