# Supplementary material for: Temperature‐ and Light‐Regulated Liquid Crystal Smart Window for Dynamic Control of Daylight and Solar Heat in All‐Weather Conditions
Source: Angew Chem Int Ed Engl. 2026 Jun 20;65(34):e7998291. doi: 10.1002/anie.7998291 (PMC13480729; doi:10.1002/anie.7998291)
Supplement: Supplementary file 1 [file ANIE-65-e7998291-s002.pdf]

Supporting Information

Temperature- and Light-Regulated Liquid Crystal Smart Window for Dynamic Control of Daylight and Solar Heat in All-Weather Conditions

Yang Zhang[1,5], Ying Wu[1], Wenxuan Liu[1,2], Xin Wang[3], Dick J. Broer[4], Ben L. Feringa[1,2*], Jiawen Chen[1*]

[1]SCNU-UG International Joint Laboratory of Molecular Science and Display, South China Normal University, Guangzhou 510006, China.

[2]Stratingh Institute for Chemistry, University of Groningen, 9747AG Groningen, The Netherlands.

[3]Institute of Carbon Neutrality, Zhejiang Wanli University, Ningbo 315100, China.

[4]Department of Chemical Engineering and Chemistry, Eindhoven University of Technology, Den Dolech 2, 5612 AZ Eindhoven, The Netherlands.

[5]International Academy of Optoelectronics at Zhaoqing, South China Normal University, Zhaoqing 526060, China.

Experimental Section

Materials: The commercially available 4-cyano-4′-octylbiphenyl (8CB), the chiral dopant S5011, and the LC monomer 1,4-bis(4-(6-acryloyloxyhexyloxy)benzoyloxy)-2-methylbenzene (HCM 009) were purchased from HCCH (Nanjing, China). The photoinitiator Irg 784 was obtained from TCI (Tokyo, Japan). The light-driven chiral dopant motor **1** was synthesized following procedures reported in our previous works, and chemicals and solvents (analytical grade) were used as obtained without further purification.

Preparation of the TLLC smart window: 8CB, motor **1**, S5011, HCM 009, and Irg 784 were mixed at 90 ºC. The mixture was injected into a glass cell with planar alignment layers, which had been prepared from glasses treated with polyvinyl alcohol (PVA). The cell gap was 20 μm, controlled by spacers of silica microspheres. After filling, the cells were exposed to green light for 30 min at 25 ºC.

Characterization: The transmittance spectra from 400 nm to 1500 nm was acquired on a UV-Vis-NIR spectrometer (Perkin Elmer Lambda 950). The transmittance data were normalized with normal glass as 100%. A deuterium lamp and a tungsten halogen lamp were used as unpolarized light sources, and no polarizing optics were employed during the measurements. Polarized optical microscope (POM, Leica DM 2700P) was utilized to investigate the LC textures. An LED lamp (Thorlabs) at 365 nm was used for the UV light sensitivity test. The power of UV light was measured using a photometer (Thorlabs PM100D) equipped with a Si photodiode sensor (S120VC). To confirm the phase transition temperature, DSC curves were measured using a Mettler Toledo DSC 1 with a rate of 5 °C/min used for both the heating and cooling ramp. Scanning electron microscopy (SEM) images were taken using a scanning electron microscope Zeiss Gemini 500 that was operated with an acceleration voltage of 5 kV.

The center selective reflection wavelength (λ) of the CLC is calculated as follows:

$$\lambda = |n \cdot p| = \left| \frac{1}{HTP_{\mathbf{1}} \cdot c_{\mathbf{1}} + HTP_{S5011} \cdot c_{S5011}} \right| \quad (1)$$

The average refractive index of the LC mixture is n = 1.57, p is the pitch calculated

from the HTP values, where the chiral dopant S5011 exhibits an HTP of -107μm^{-1} and c is the weight percentage concentration of the chiral dopant.

Energy-saving Potential Simulation: Software EnergyPlus was used to estimate the energy consumption of the specimens in this work. EnergyPlus evaluates a building's energy performance by considering the heat exchange between its internal and external environments across various surfaces, including walls, roofs, windows, and doors. Factors affecting the internal environment include the HVAC system, lighting, plug loads (e.g., office equipment, refrigerators), and ventilation. External boundary conditions, such as temperature and sunlight, were determined using the Typical Meteorological Year (TMY), while the simulation followed relevant building energy efficiency standards, incorporating typical occupancy schedules, internal heat gains from lighting and equipment, and standard HVAC system settings. The simulation uses a 10-story office building with a window area of 2133.5 m^2, and a window-to-wall ratio of 50%. The performance of the sample was controlled using the built-in Energy Management System (EMS) in EnergyPlus. The total floor area of the building served as the reference for calculating energy savings per unit area.

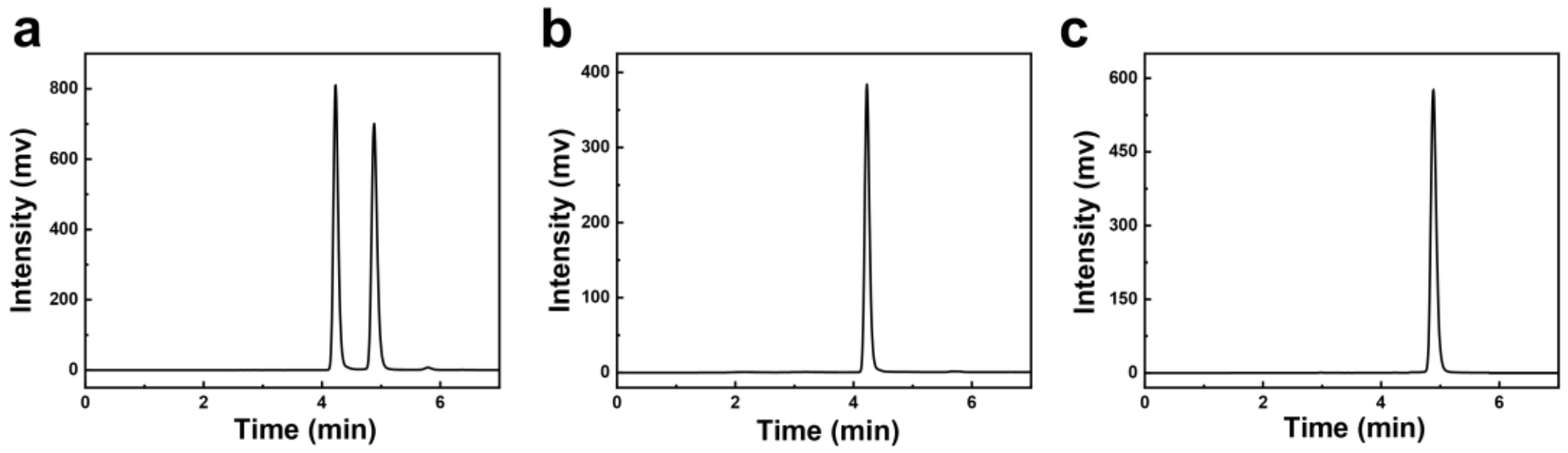

Figure S1. HPLC spectra of (a) racemic **1**, (b) (*S*)-**1** and (c) (*R*)-**1**. The separation and characterization were performed by chiral HPLC using chiral stationary phase (Chiralpak IG, hexane: DCM 75: 25) with a flow rate of 1.0 mL/min.

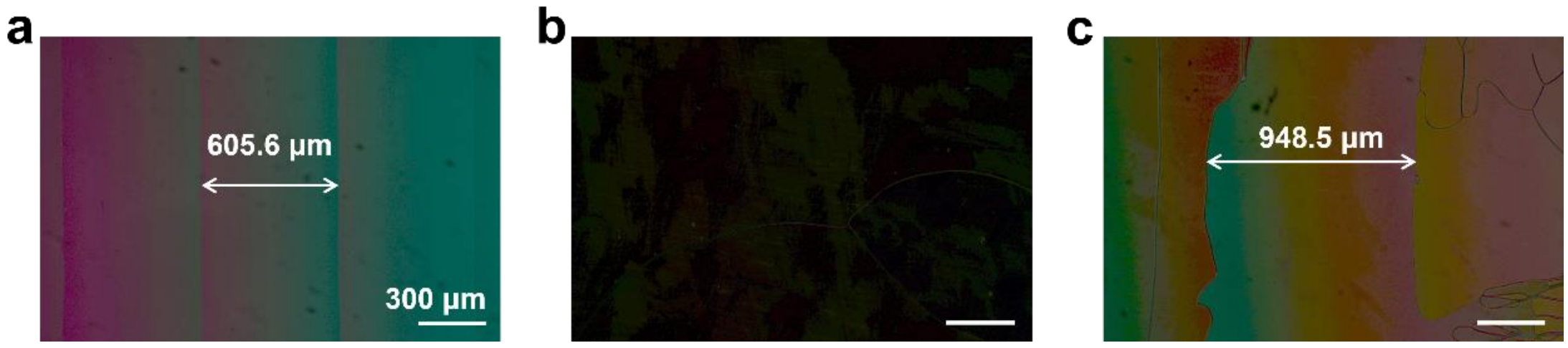

Figure S2. Distances between the disclination lines of chiral dopant **1** in 8CB (a) in the initial state, (b) unwinding state and (c) after UV light irradiation, observed from a wedge cell.

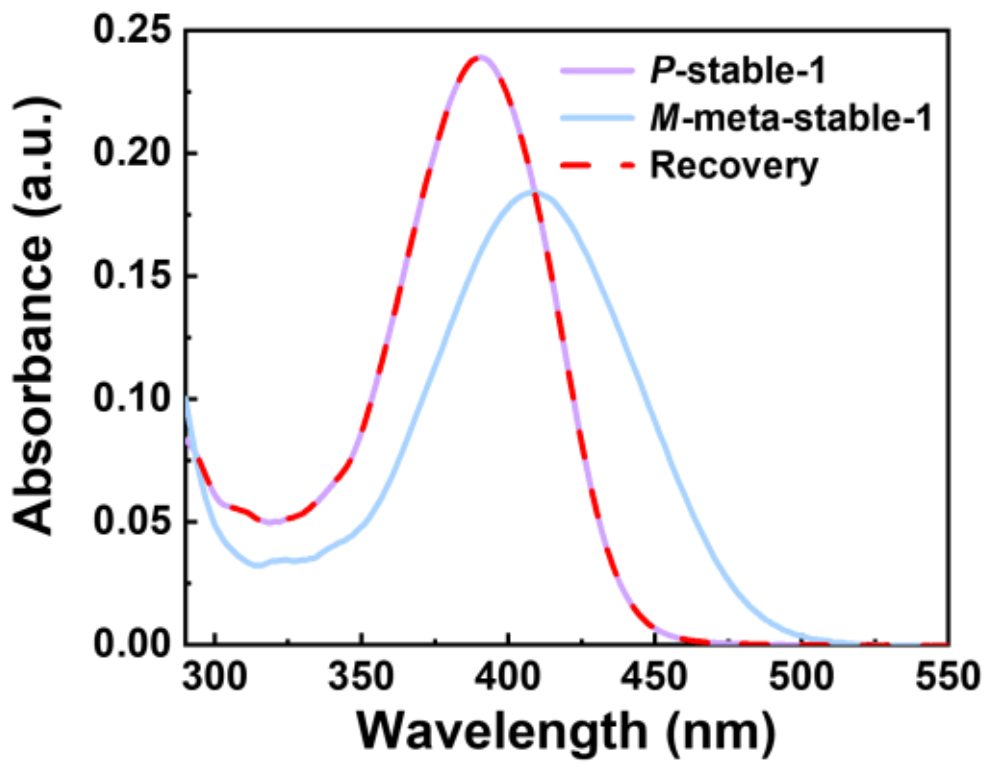

Figure S3. UV-vis spectra absorption spectra of motor **1** (c = 1×10^{-5} M) at 25 ^{o}C (in THF).

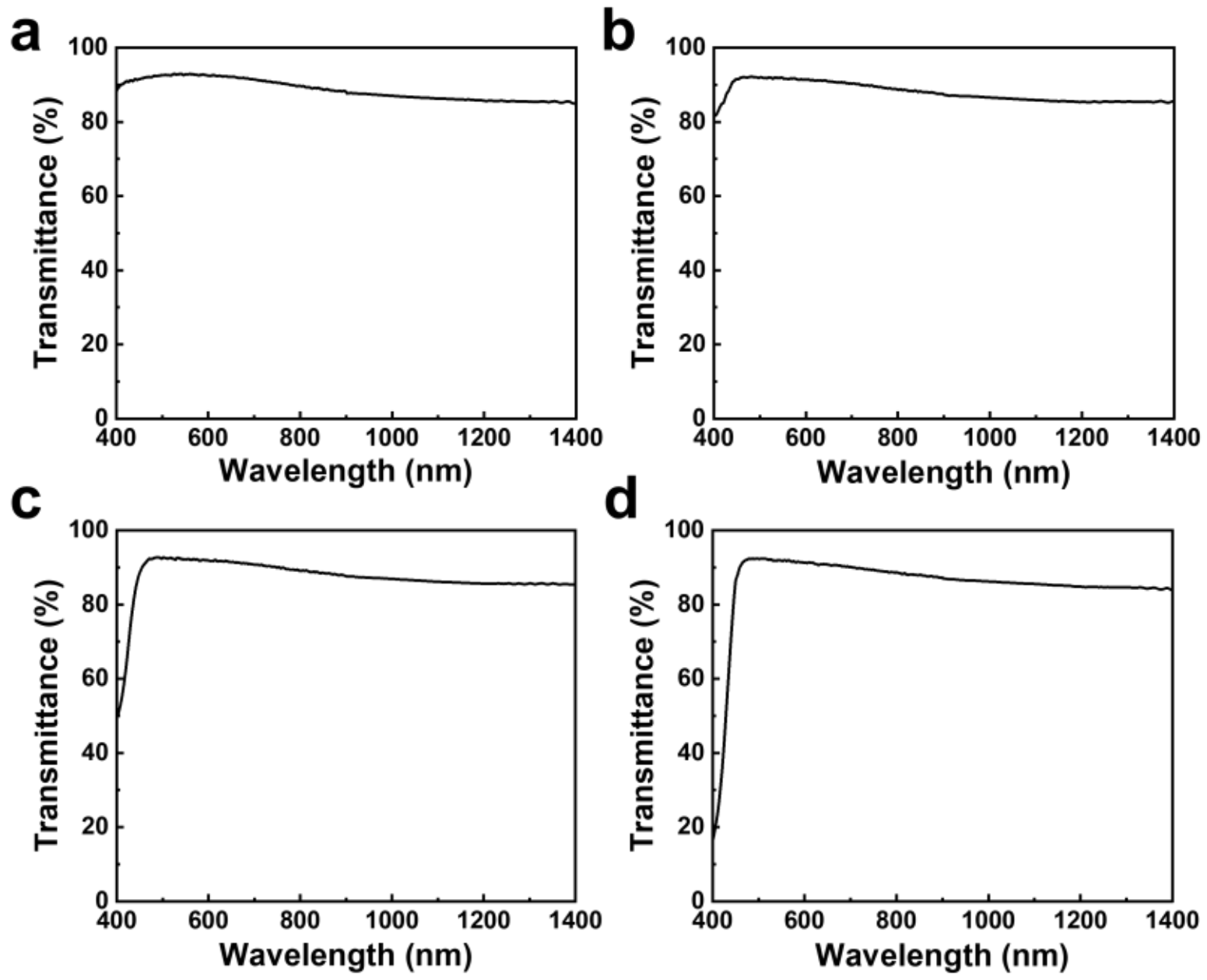

Figure S4. Transmittance spectrum of LC mixtures of 8CB with (a) 0 wt%, (b) 0.1 wt%, (c) 0.3 wt% and (d) 0.5% chiral dopant **1**.

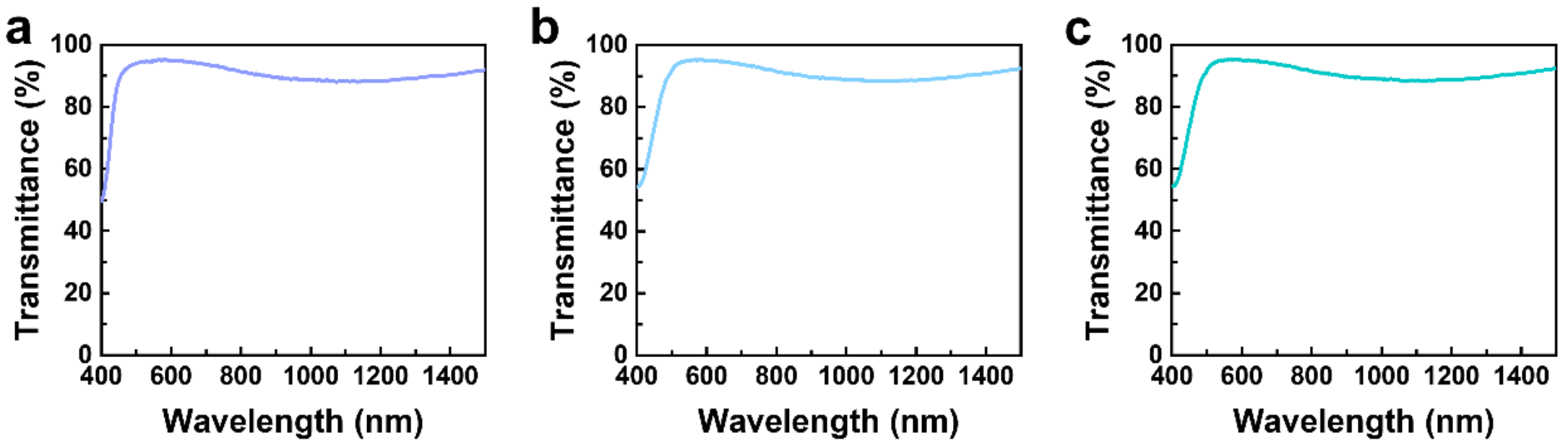

Figure S5. Transmittance spectrum of the TLLC smart window in the SmA phase under UV light irradiation with intensities of (a) 0.0%, (b) 0.3% and 1.0% mW/cm^2

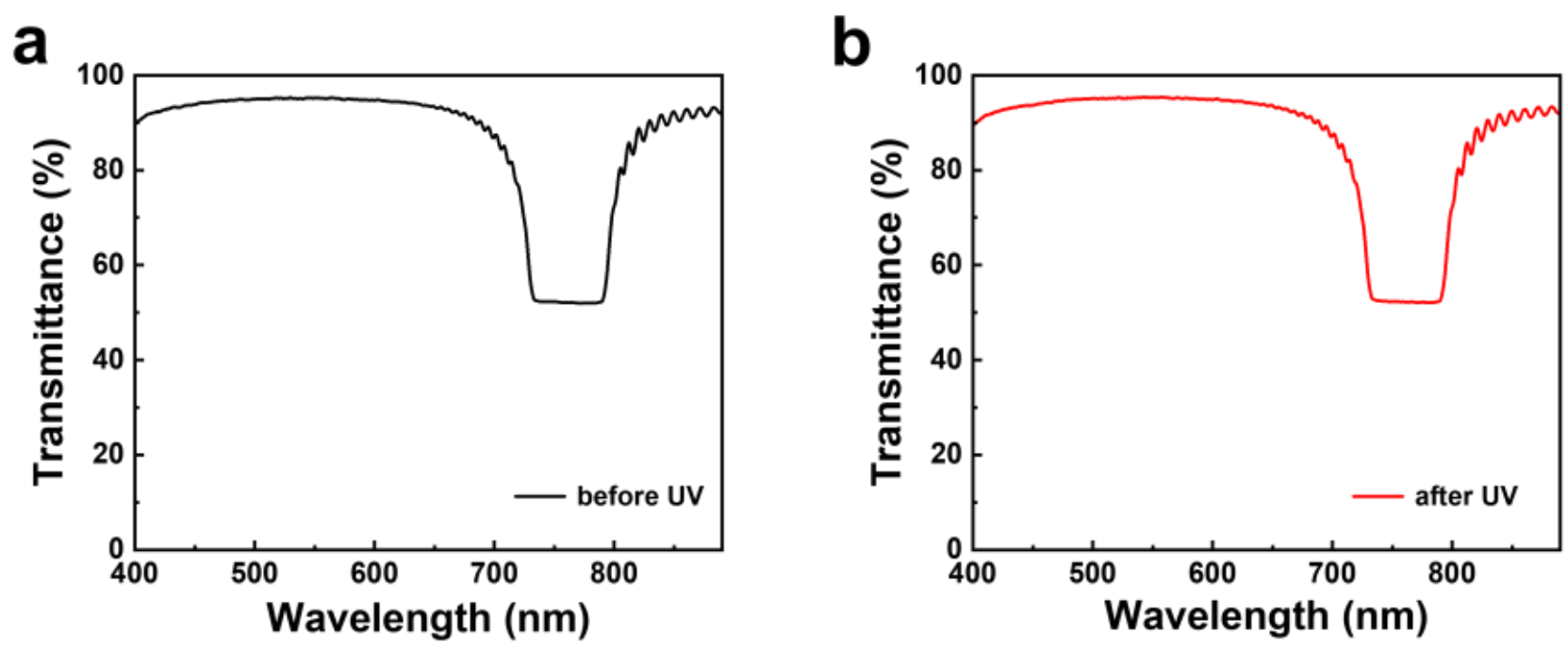

Figure S6. Transmittance spectrum of the 8CB/S5011 mixture measured (a) before and (b) after exposure to UV light.

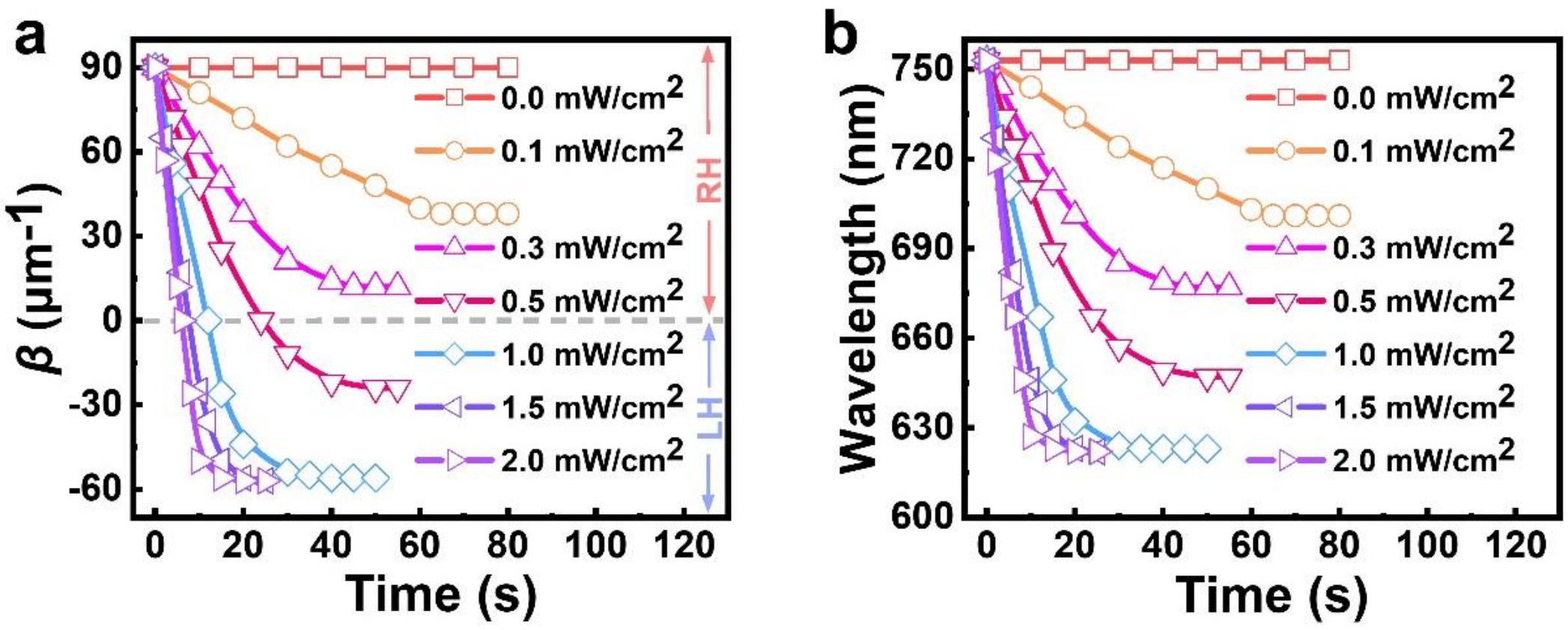

Figure S7. (a) The variation in the helical twisting power (HTP) of molecular motor **1** and (b) the reflective wavelength of the TLLC window under different UV exposure intensities.

Molecular motor **1** exhibits a high helical twisting power (HTP), which enables a larger range of reflective band tuning. In addition, the high HTP value allows for a lower concentration of the molecular motor that is used, thereby minimizing its intrinsic absorption in the region below 400 nm and maintaining the optical transparency of the device (Figure S4). Furthermore, molecular motor **1** possesses good photo- and thermal stability, which is crucial for achieving reversible optical switching behavior. Meanwhile, we investigated the change of the HTP value of molecular motor **1** as a function of irradiation time under fixed light intensity in Figure S7a. With increasing exposure time, the HTP value reaches a steady state within seconds, indicating that the kinetic process of molecular motor **1** is able to drive the pitch adjustment and consequently realize the shift of reflection band.

Regarding the co-dopant S5011, its concentration was set to 2.2 wt% because we want to adjust the initial reflective center of the system to the near-infrared region (approximately 755 nm), which facilitates the modulation of solar heat while minimizing any significant impact on transmittance of visible light. Upon light stimulation, the isomerization of the molecular motor alters the helical pitch of the system, allowing the reflection band to further shift into the visible region, thereby generating a distinct color change that serves as a visual indicator of the change of operating state.

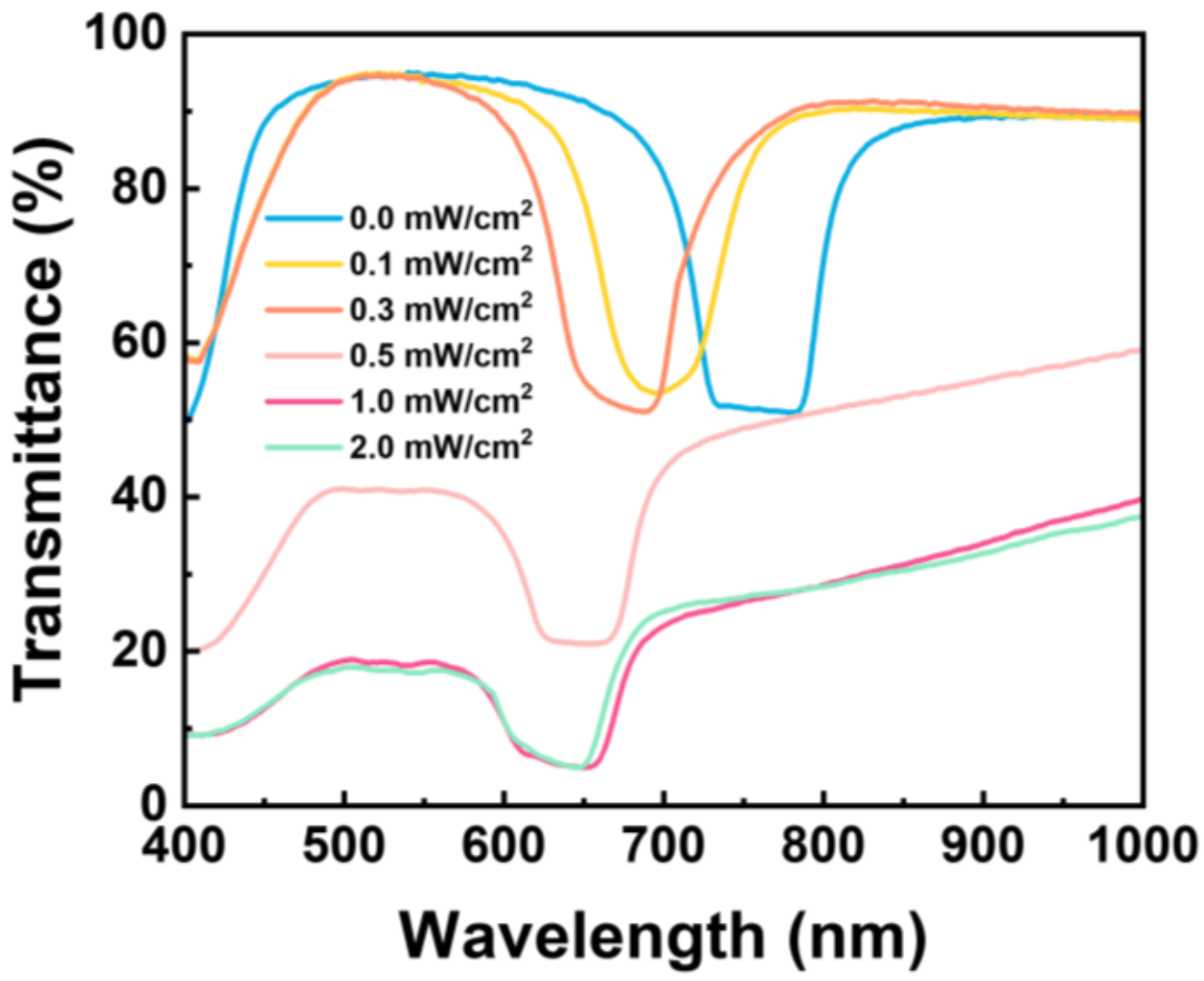

Figure S8. Transmittance spectrum of TLLC smart window under different UV irradiation intensities in the N* phase

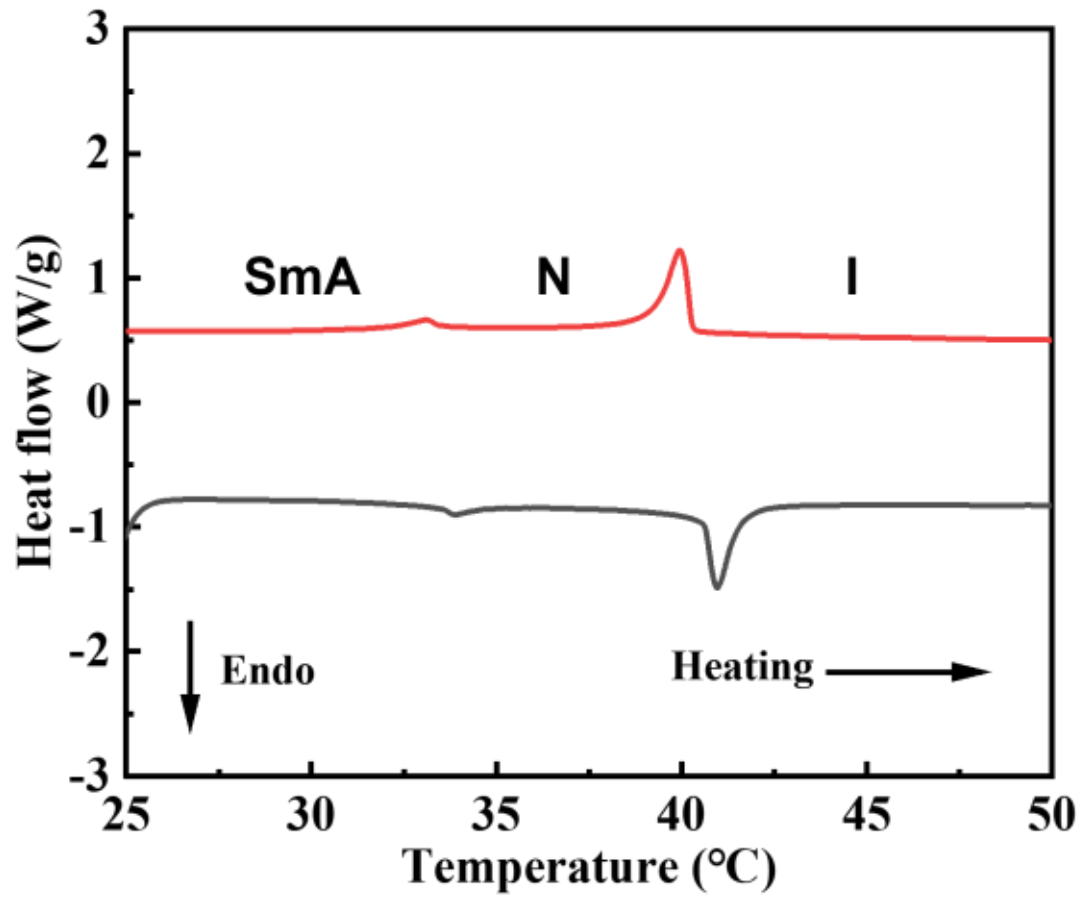

Figure S9. Differential scanning calorimetry curves of liquid crystal 8CB

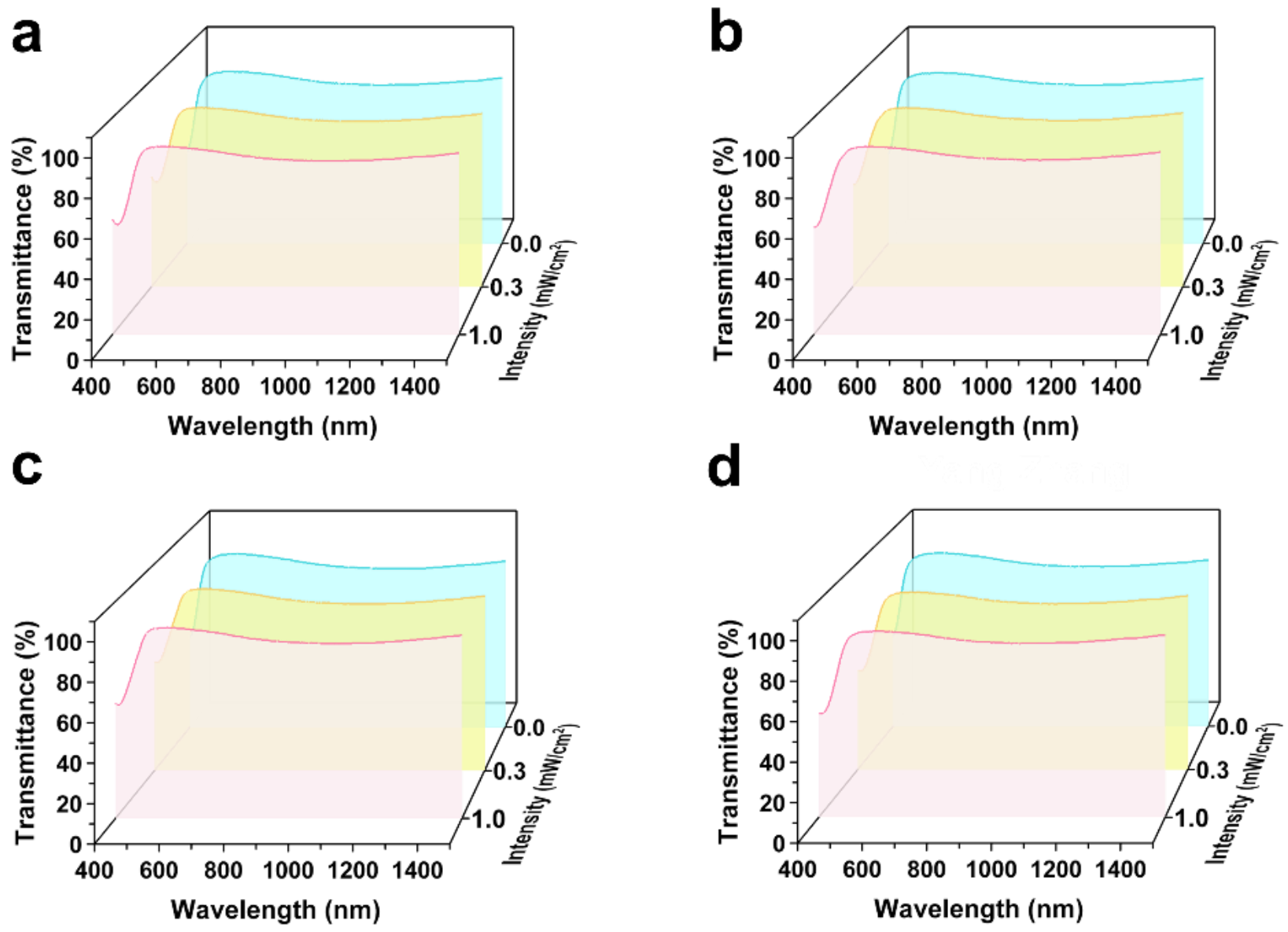

Figure S10. Transmittance spectrum of TLLC smart windows prepared with (a) 0.0 wt%, (b) 1.0 wt%, (c) 3.0wt% and (d) 5.0 wt% monomer concentrations under different UV irradiation intensities in the SmA phase

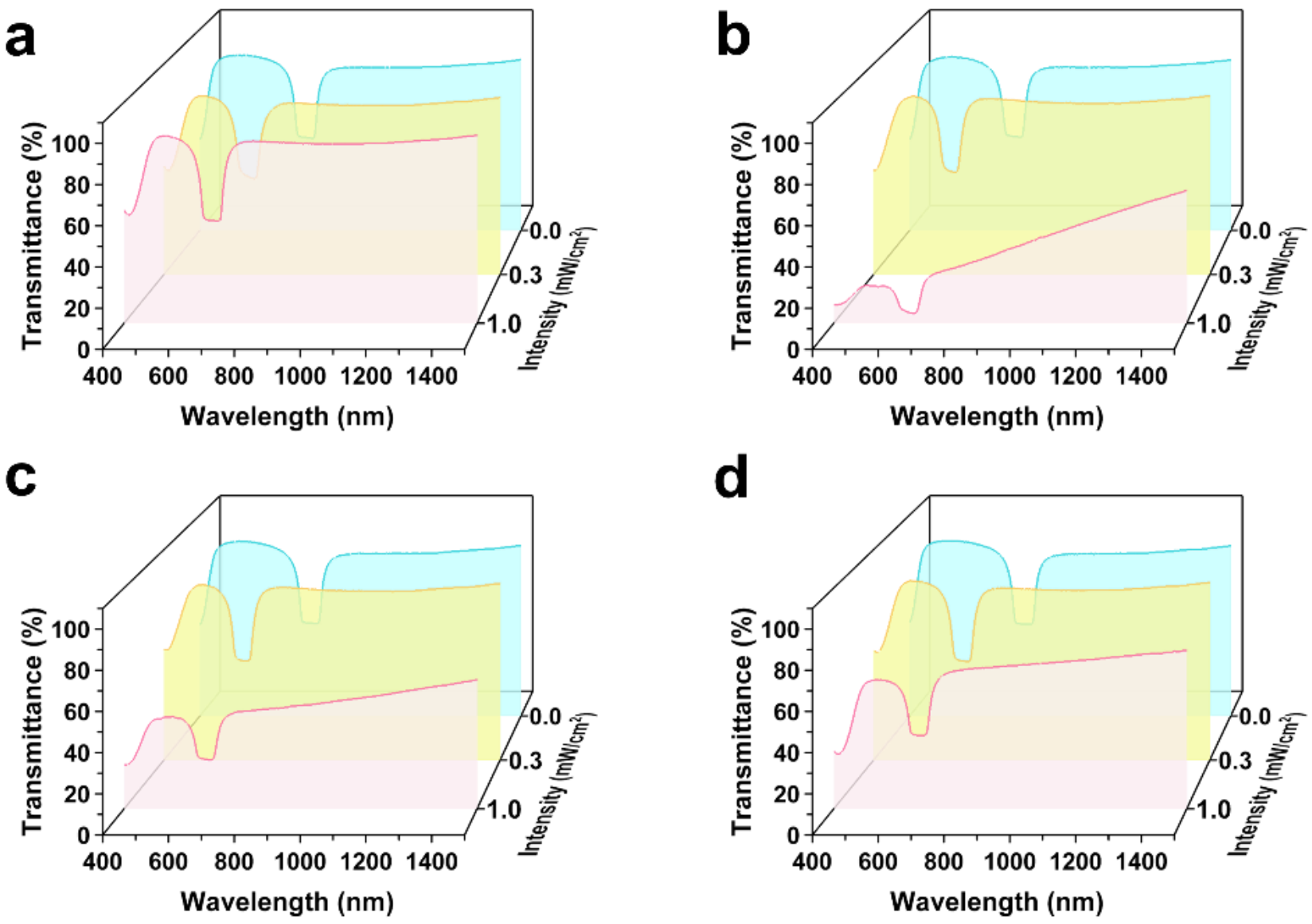

Figure S11. Transmittance spectrum of TLLC smart windows prepared with (a) 0.0 wt%, (b) 1.0 wt%, (c) 3.0wt% and (d) 5.0 wt% monomer concentrations under different UV irradiation intensities in the N* phase

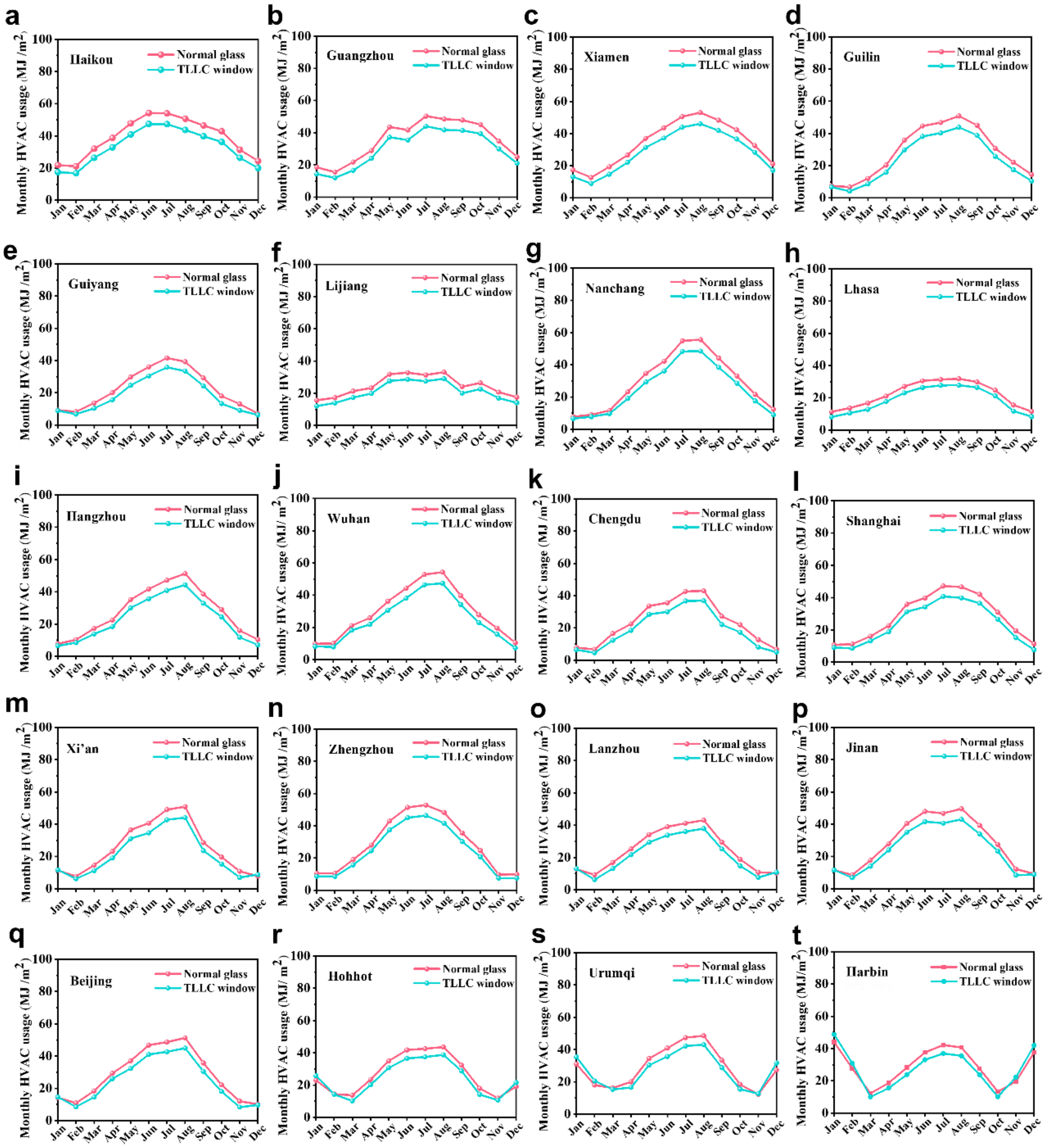

Figure S12. (a-t) Monthly HVAC energy consumption of normal glass and TLLC smart window in Haikou, Guangzhou, Xiamen, Guilin, Guiyang, Lijiang, Nanchang, Lhasa, Hangzhou, Wuhan, Chengdu, Shanghai, Xi'an, Zhengzhou, Lanzhou, Jinan, Beijing, Hohhot, Urumqi and Harbin.

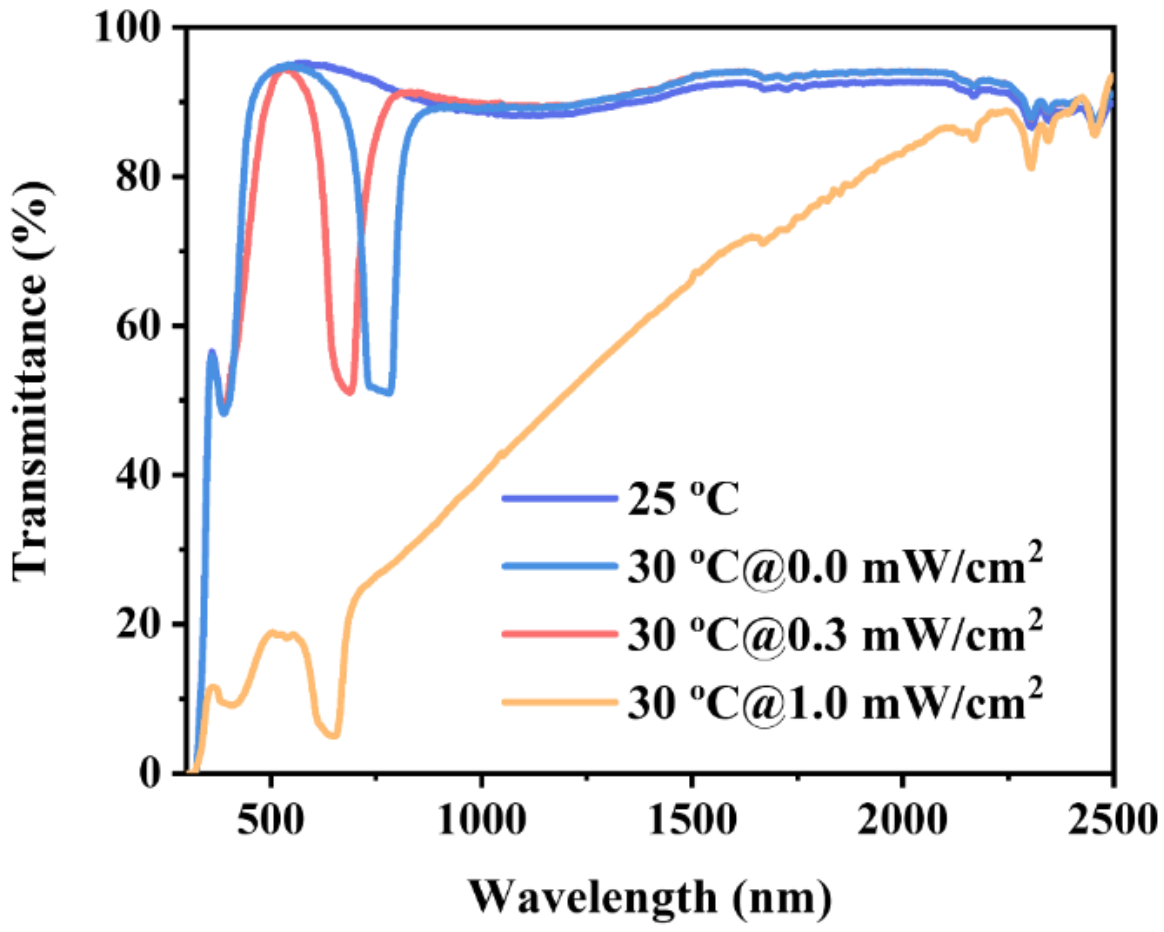

Figure S13. Transmittance spectra of TLLC window in different conditions

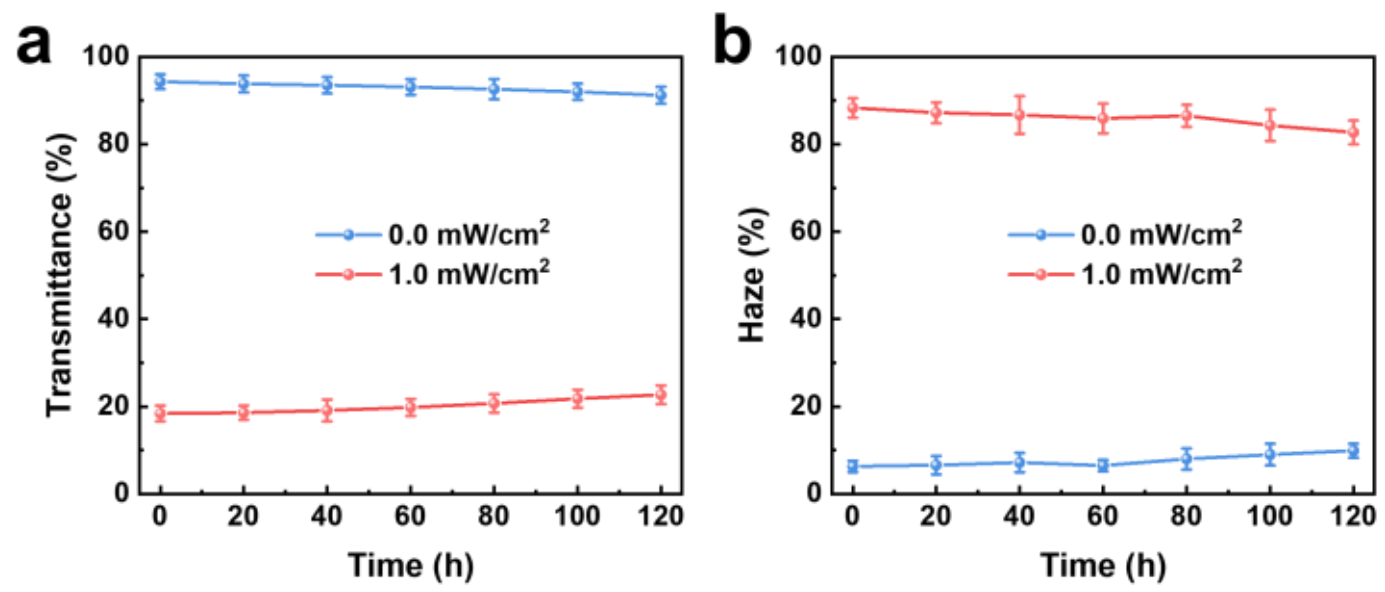

Figure S14. Transmittance and Haze of the TLLC window after 120 h UV irradiation

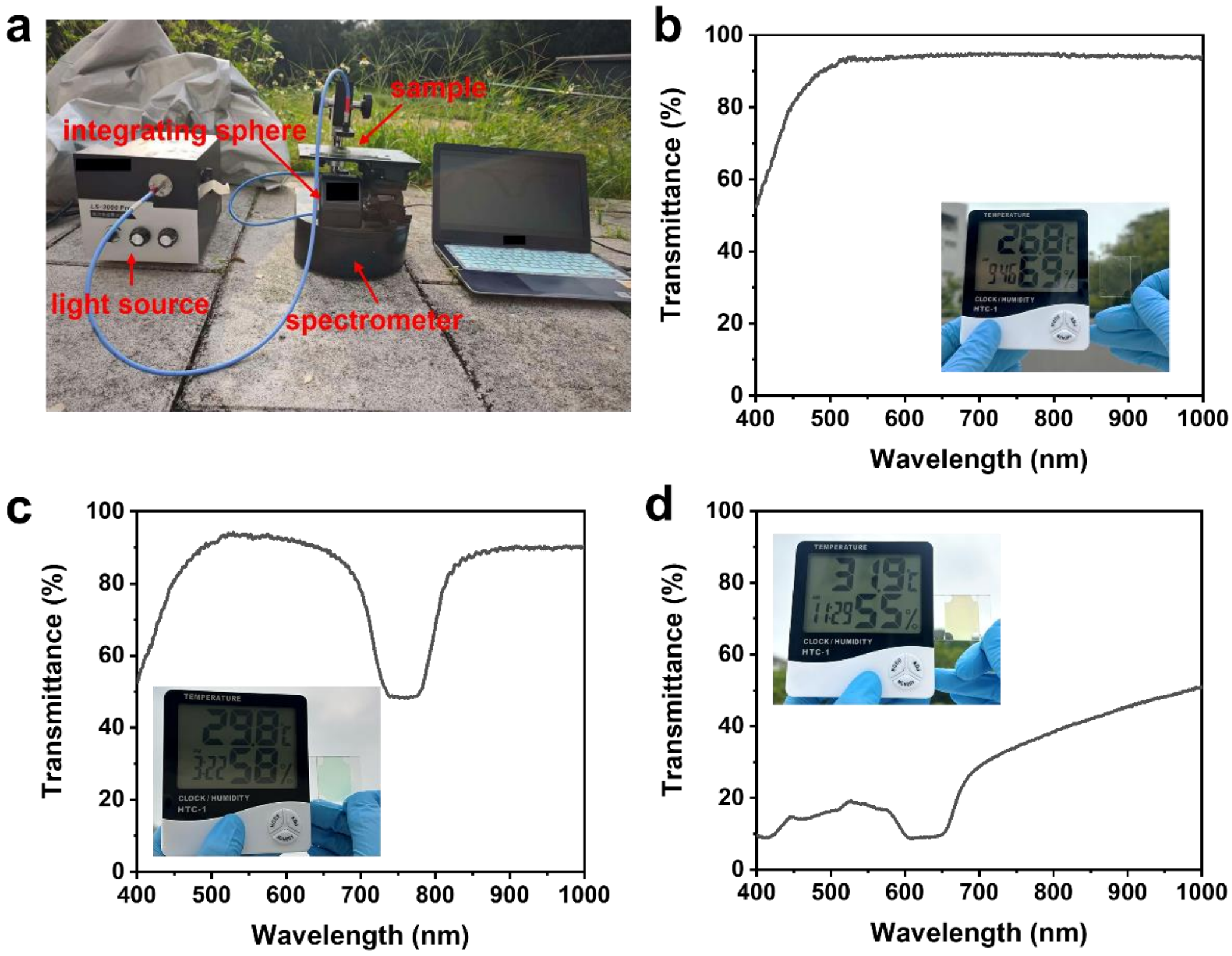

Figure S15. Outdoor in situ optical measurement of the TLLC window under different weather condition. (a) Set-up. (b) At 27 °C. (c) Cloudy day at 30 °C. (d) Sunny day at 32 °C.

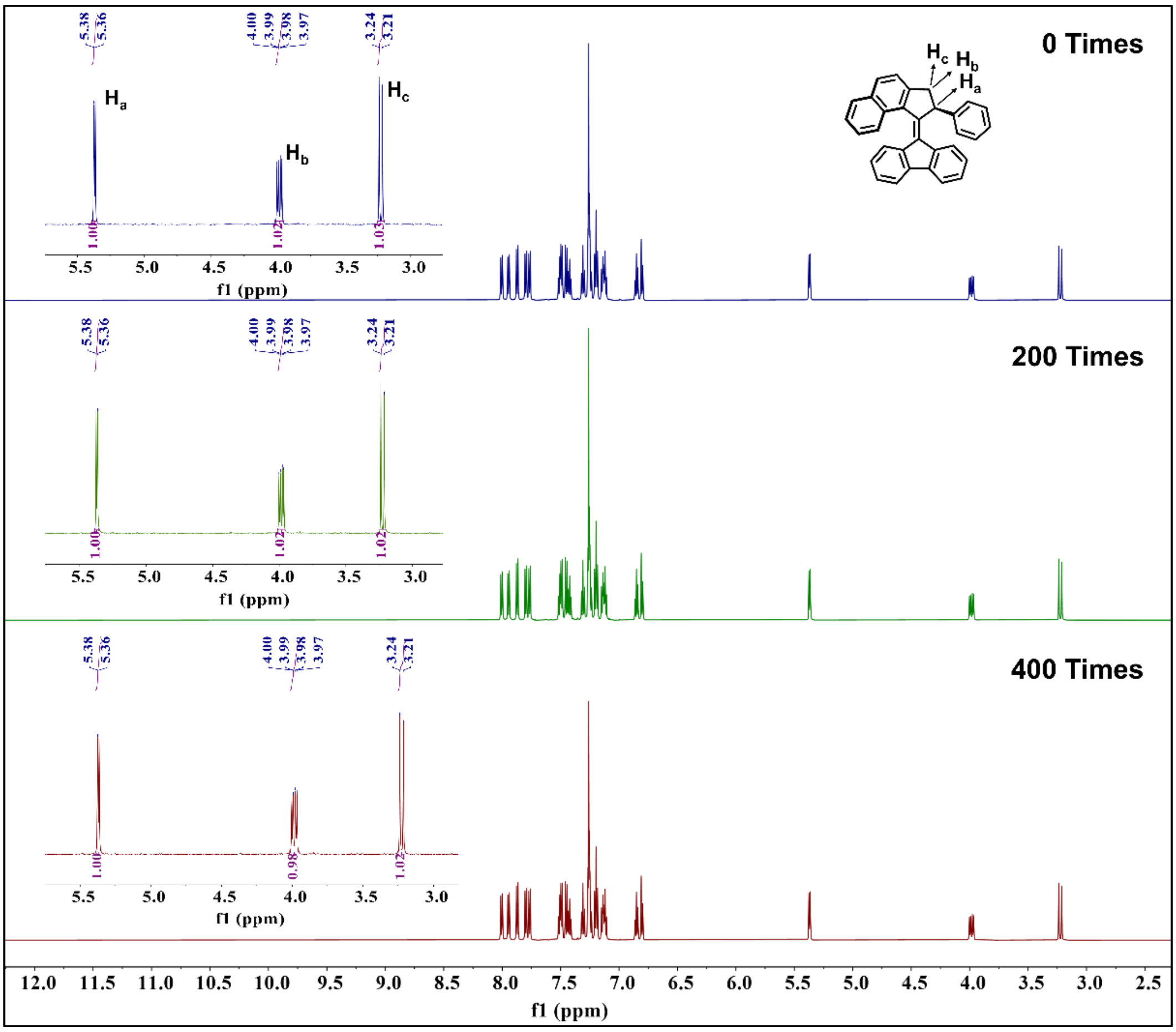

Figure S16. ^{1}H NNR spectra (25 °C, $CDCl_3$, 500 MHz) of molecular motor **1** before and after UV irradiation test.

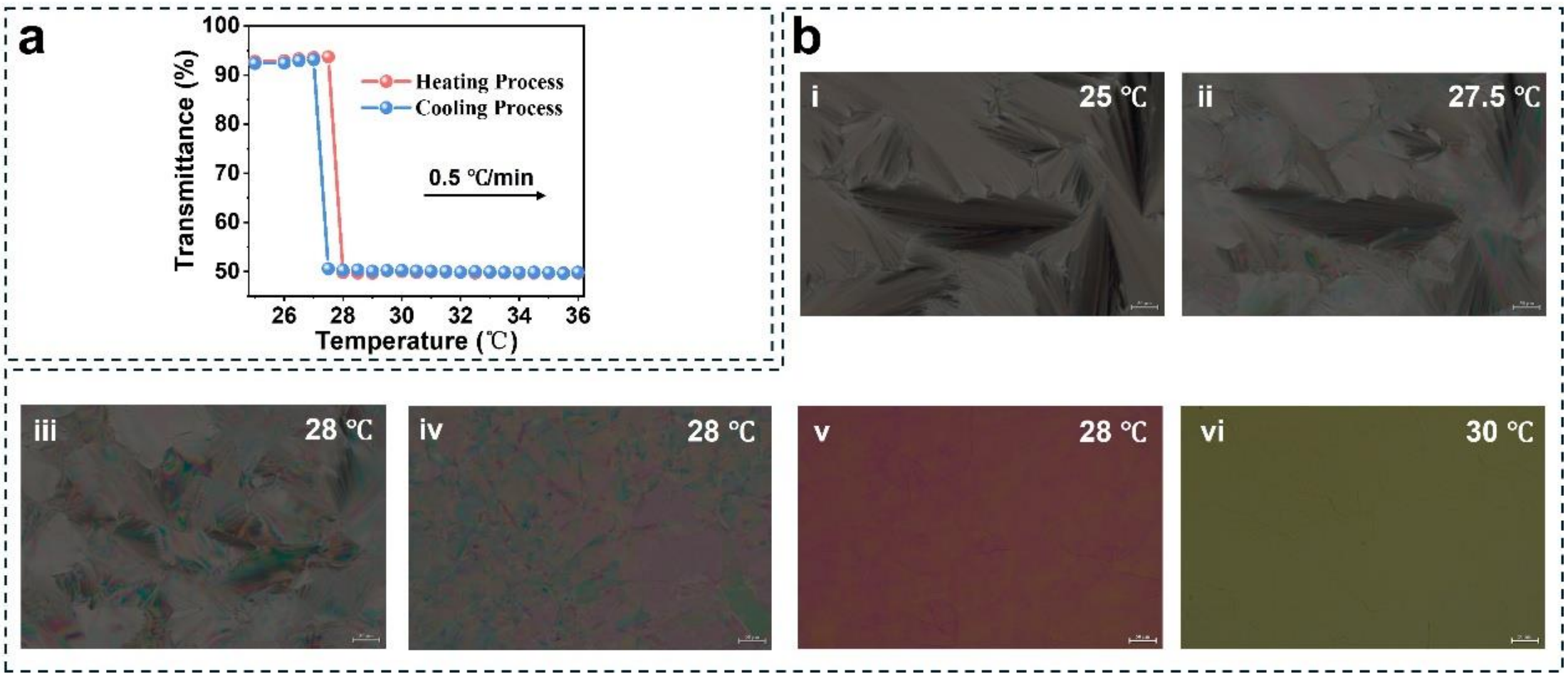

Figure S17. (a) Changes of transmittance of the TLLC window during heating and cooling process at a rate of 0.5 °C/min. (b) POM images at different temperatures.

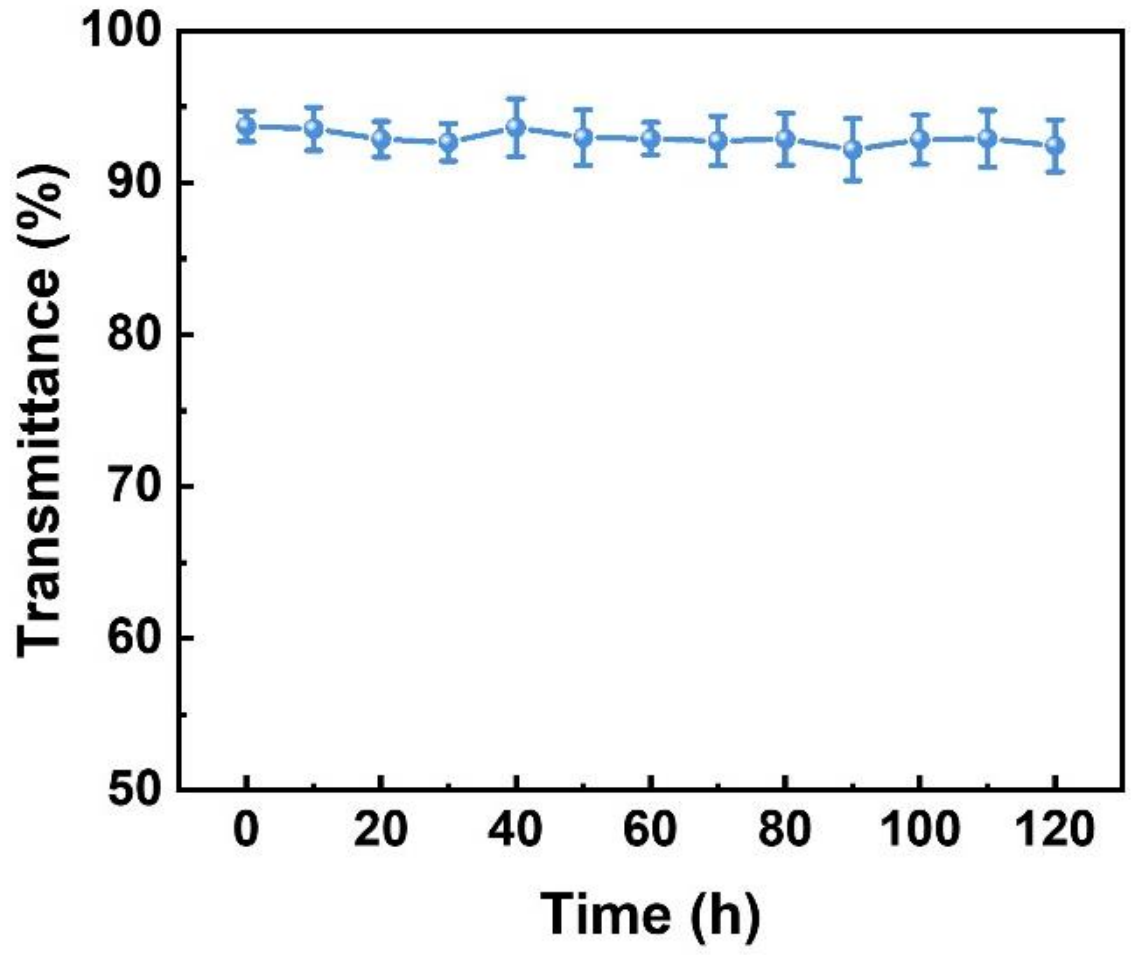

Figure S18. Transmittance spectra of TLLC window at 80 ℃ for 120 h.

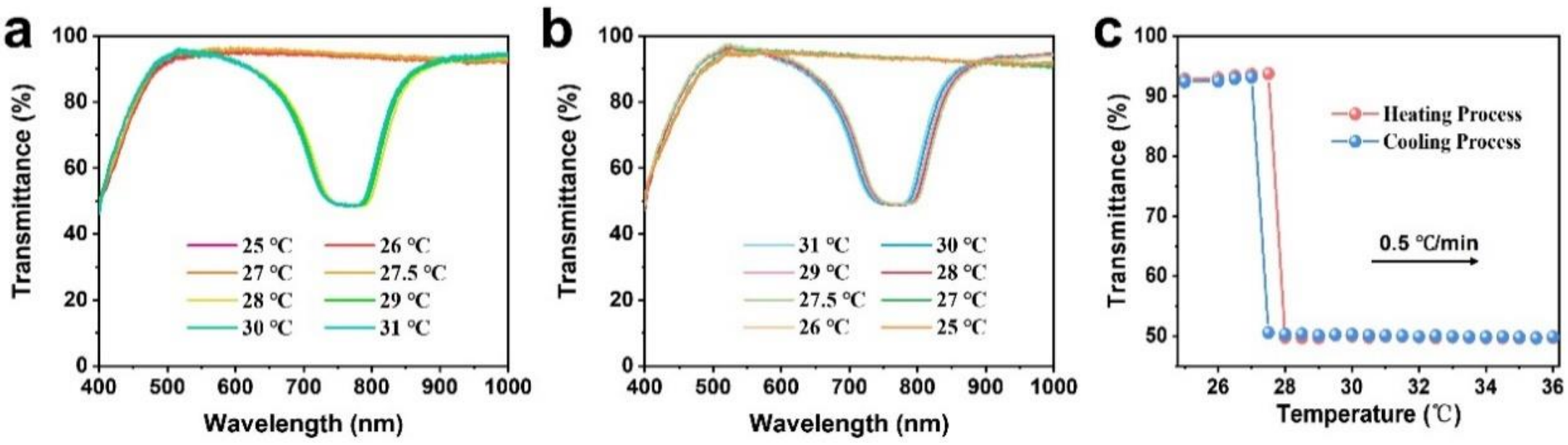

Figure S19. Changes of transmittance of TLLC window upon heating and cooling.

The thresholds T > 28 °C and UV intensity > 0.3 mW/cm² used in the present study were determined based on experimentally observed state transition behavior. Through DSC and in situ transmittance measurements and photo-response tests during heating and cooling, we found that the sample exhibits a distinct optical state transition around 28 °C, which was thus defined as the thermal-responsive threshold. Meanwhile, under conditions where the temperature was maintained above the phase transition range, the optical response was tested as a function of UV intensity, and it was found that the device shown a state switch when the UV intensity exceeded 0.3 mW/cm² and thus this value was defined as the photo-responsive threshold.

The operational state of glazing in practical buildings is influenced not only by ambient temperature and solar irradiation but also by factors such as wind speed, intermittent cloud cover and shading conditions. Therefore, these two thresholds should be more accurately understood as experimental triggering conditions for the current device with the present formulation under the tested conditions, rather than as fixed parameters for all climatic scenarios. Meanwhile, our supplementary heating and cooling tests (Figure S19) show that the system exhibits only a small thermal hysteresis (approximately 0.5 °C) near the phase transition, and the transient state remains for only a short duration. The above data suggests that in response to short-term weather fluctuations (e.g., intermittent cloud cover), the device is more likely to exhibit only brief, limited variations rather than prolonged instability.

Table S1 Key parameters of the TLLC smart window

**State**	**25 ºC**	**30 ºC@0.0mW/cm²**	**30 ºC@0.3mW/cm²**	**30 ºC@1.0mW/cm²**
T_{lum}	90.8%	90.7%	86.0%	15.6%
T_{NIR}	91.3%	86.5%	91.6%	42.0%
T_{sol}	88.1%	84.4%	86.8%	29.5%
SHGC	0.84	0.82	0.83	0.49

Table S2 Comparison of key parameters of TLLC window and exiting smart windows

Categories	Trigger mode	ΔT_{sol}	ΔT_{lum}	ΔT_{NIR}	Response time (t_{on})	Durability	Ref
Liquid crystal	temperature & light	58.6%	75.2%	49.3%	T: 5 s<br>UV: 18 s	1000 fatigue cycles	This work
	light	16.5%	17.8%	14.8%	300 s	100 fatigue cycles	[1]
	electric (12 V)	70.2%	74.9%	–	20 ms	500 fatigue cycles	[2]
	electric & temperature	13.9%	54.8%	–	9 ms	Not mentioned	[3]
VO_2	temperature	65.7%	–	46.5%	–	100 fatigue cycles	[4]
	temperature	20.0%	19.7%	–	–	23 years	[5]
	temperature	8.8%	2.0%	–	150 s	Not mentioned	[6]
Hydrogel	temperature	58.4%	68.4%	–	–	500 fatigue cycles	[7]
	temperature	57.2%	95.2%	44.3%	50 s	100 fatigue cycles	[8]
Perovskite	temperature	23.7%	54.9%	–	1-4 min	50 fatigue cycles	[9]
	temperature	25.4%	53.1%	0.0%	a few minutes	Not mentioned	[10]
Ionic liquid	temperature	36.1%	41.5%	–	165 s	600 fatigue cycles	[11]
	temperature	27.3%	25.1%	–	–	Not mentioned	[12]
WO_3	temperature	44.0%	39.0%	–	30 min	50 fatigue cycles	[13]
	temperature	–	40.0%	–	30 min	–	[14]

[1] W. Meng, Y. Gao, X. Hu, L. Tan, L. Li, G. Zhou, H. Yang, J. Wang, L. Jiang, *ACS applied materials & interfaces* **2022**, 14, 28301.

[2] Z. Zhang, X. He, L. Zhang, J. Xu, B. Yuan, C. Chen, C. Zou, Q. Wang, Y. Gao, M. Yu, *Chemical Engineering Journal* **2024**, *479*, 147668.

[3] F. Jing, Z. Zhang, L. Zhang, F. Zhou, J. Qin, C. Zou, Y. Gao, M. Yu, H. Yang, *Small* **2025**, *21*, e06780.

[4] Y. Feng, W. Ma, H. Li, M. Yang, Y. Yu, S. Liu, X. Zeng, F. Huang, Y. Yang, Z. Li, *ACS applied materials & interfaces* **2023**, *15*, 5836.

[5] X. Zhou, Y. Meng, T. D. Vu, D. Gu, Y. Jiang, Q. Mu, Y. Li, B. Yao, Z. Dong, Q. Liu, *Journal of Materials Chemistry A* **2021**, *9*, 15618.

[6] Q. Hao, W. Li, H. Xu, J. Wang, Y. Yin, H. Wang, L. Ma, F. Ma, X. Jiang, O. G. Schmidt, *Advanced materials* **2018**, *30*, 1705421.

[7] C. Lin, J. Hur, C. Y. Chao, G. Liu, S. Yao, W. Li, B. Huang, *Science advances* **2022**, *8*, eabn7359.

[8] L. Xie, X. Wang, X. Zou, Z. Bai, S. Liang, C. Wei, S. Zha, M. Zheng, Y. Zhou, O. Yue, *Small* **2023**, *19*, 2304321.

[9] S. Liu, Y. W. Du, C. Y. Tso, H. H. Lee, R. Cheng, S. P. Feng, K. M. Yu, *Advanced Functional Materials* **2021**, *31*, 2010426.

[10] Y. Zhang, C. Tso, J. S. Iñigo, S. Liu, H. Miyazaki, C. Y. Chao, K. M. Yu, *Applied energy* **2019**, *254*, 113690.

[11] H. Wang, B. Zhang, J. Wang, Y. Lu, T. Qi, X. Tian, M. Wang, Z. Qu, W. Zhou, F. Sun, *Small* **2025**, *21*, 2504399.

[12] F. Xu, X. Cao, J. Zhu, G. Sun, R. Li, S. Long, H. Luo, P. Jin, *Materials Letters* **2018**, *222*, 62.

[13] W. Meng, A. J. Kragt, X. Hu, J. S. van der Burgt, A. P. Schenning, Y. Yue, G. Zhou, L. Li, P. Wei, W. Zhao, Y. Li, J. Wang, L. Jiang, *Advanced Function Materials* **2024**, *34*, 2402494.

[14] W. Meng, J. Wang, L. Jiang, *Responsive Materials* **2024**, *2*, e20240001.
